# Public health and chronic low chlordecone exposure in Guadeloupe, Part 1: hazards, exposure-response functions, and exposures

**DOI:** 10.1186/s12940-016-0160-x

**Published:** 2016-07-12

**Authors:** Vincent Nedellec, Ari Rabl, William Dab

**Affiliations:** Consultant on Environmental risks and health safety, 23, rue André Masséna, 83000 Toulon, France; Retired from Ecole des Mines/ARMINES, Paris, Consultant on Environmental Impacts, 6 av. Faidherbe, 91440 Bures sur Yvette, France; Conservatoire National des Arts et Métiers (CNAM), 292, rue Saint Martin, 75141 Paris cedex 03, France

**Keywords:** Chlordecone, Low-dose, Mode of action, Key-events, Exposure-response function, Risk assessment, Non-mutagenic agent, Endocrine disrupter

## Abstract

**Background:**

Inhabitants of Guadeloupe are chronically exposed to low dose of chlordecone via local food. The corresponding health impacts have not been quantified. Nevertheless the public authority implemented an exposure reduction program in 2003. We develop methods for quantifying the health impacts of chlordecone and present the results in 2 articles: 1. hazard identification, exposure-response functions (ERF) and exposure in Guadeloupe, 2. Health impacts and benefits of exposure reduction. Here is the first article.

**Methods:**

Relevant data are extracted from publications searched in Medline and Toxline. Available knowledges on mode of action and key-event hazards of chlordecone are used to identify effects of chlordecone that could occur at low dose. Then a linear ERF is derived for each possible effect. From epidemiological data, ERF is the delta relative risk (RR-1) divided by the corresponding delta exposure. From animal studies, ERF is the benchmark response (10 %) divided by the best benchmark dose modeled with BMDS2.4.0. Our goal is to obtain central values for the ERF slopes, applicable to typical human populations, rather than lower or upper bounds in the most sensitive species or sex.

**Results:**

We derive ERFs for 3 possible effects at chronic low chlordecone dose: cancers, developmental impairment, and hepatotoxicity. Neurotoxicity in adults is also a possible effect at low dose but we lack quantitative data for the ERF derivation. A renal toxicity ERF is derived for comparison purpose. Two ERFs are based on epidemiological studies: prostate cancer in men aged >44y (0.0019 per μg/L_blood_) and altered neurodevelopment in boys (−0.32 QI_point_ per μg/L_cord-blood_). Two are based on animal studies: liver cancer (2.69 per mg/kg/d), and renal dysfunction in women (0.0022 per mg/kg/d).

**Conclusion:**

The methodological framework developed here yields ERFs for central risk estimates for non-genotoxic effects of chemicals; it is robust with regard to models used. This framework can be used generally to derive ERFs suitable for risk assessment and for cost-benefit analysis of public health decisions.

**Electronic supplementary material:**

The online version of this article (doi:10.1186/s12940-016-0160-x) contains supplementary material, which is available to authorized users.

## Background

The inhabitants of the French West Indies are chronically exposed to low doses of chlordecone via local food. The corresponding public health issues have not been satisfactorily quantified because of the lack of an appropriate risk assessment method. Nonetheless, several million euros have been invested every year since 2003 for the reduction of exposure in Martinique and Guadeloupe [1–3]. Official investigations had revealed that 1.3 % of adults in Guadeloupe were exposed above the “no effect threshold dose” (the reference dose RfD) of 0.5 μg/kg/d [4]. Exposure to chlordecone seems more common in children 3 to 5 years old than in adults, with up to 18.5 % above RfD in Martinique [5]. These estimates of ingestion exposures are based on food consumption survey data matched with the results of chlordecone measurements in food made for the screening and control program. The main activities of the control program are to withdraw food from the market and water from the distribution system when chlordecone is above the limit values [6]. However, some polluted food from individual gardens could be eaten by the owners or the relatives and friends because they escape the market control system.

The RfD is based on a chronic animal study that observed an increase in kidney damage (glomerulosclerosis) in female Wistar rats [7]. However, monitoring of poisoned workers manufacturing chlordecone in the USA (Hopewell and Baltimore) revealed no renal function impairment [8, 9]. In addition, a recent study showed that only mouse strains predisposed to auto immune diseases developed kidney lesions after ingesting chlordecone [10]. Accordingly, the hazards of chlordecone in people with exposure above this RfD are uncertain, and the results of exposure studies are difficult to translate into public health decisions. In particular, they do not provide answers to the following questions:

- What effect will occur in persons exposed above RfD: that observed at the lowest dose tested in animals, other effects known at some higher doses, or even the effect of another ubiquitous pollutant that is enhanced by chlordecone?
- If exposure exceeds the RfD what is the likelihood that an effect occurs?
- Are we sure that there will be no effect from exposures below RfD?

Several epidemiological studies have recently been carried out in Guadeloupe. The results show that chronic low exposure to chlordecone, mainly below the value of the RfD, is correlated with an increased risk of prostate cancer in men over 45 years [11] and with impaired neurobehavioral development in the young child [12, 13]. Other effects were investigated but were not significantly associated with low exposure to chlordecone: change in sperm quality [14, 15], and the rate of circulating hormones in men [16]. No studies looked at possible kidney damage, the critical effect found in laboratory animals [17, 18] nor at liver cancer which is significantly increased in a chronic animal study by the NCI [19]. The latter is used by the US-EPA to develop a cancer slope factor. In epidemiological studies exposure was estimated via blood chlordecone concentration (Cl-b).

Another concern for chlordecone health risk assessment is that we do not know sufficiently well the correspondence between Cl-b and the dose by ingestion expressed as mg/kg_BW_. First, an ad hoc study in Guadeloupe published in 2010 found that exposure data based on food consumption combined with food concentration of chlordecone are poorly correlated (R^2^ = 0.20) with blood chlordecone [20]. Second, there was no PBPK (physiologically based pharmacokinetic) model available at the time of the literature search that could help to convert a dose by ingestion into a blood concentration of chlordecone.

Health risk assessment cannot be quantitative without a quantitative relationship between exposure and response. They are commonly available for carcinogenic effects but not for other effects that have a non-genotoxic mode of action. To quantify the public health impacts of chlordecone in Guadeloupe exposure-response functions (ERFs) are necessary. Following the recommendations of the Silver Book [21], we assume that non-genotoxic effects can occur at low doses if the mode of action has been found to be active at low doses and is shared by a frequent human disease or a ubiquitous chemical. In that case exposure-response functions, similar to those for genotoxic carcinogens, can be derived for non-genetoxic effects. Our objective is to develop a methodological framework based on: 1. the selection of potential hazards from low dose exposures; 2. deriving ERFs from human or animal data; 3. evaluation of health and economic impacts; 4. compare costs of the exposure control program with public health gains resulting from the decrease in population exposure. Because of their length, methods and results are described in 2 separate articles. In this first article, the objectives are: to identify and select the hazard of low dose chlordecone to derive the corresponding ERF and to find population exposure data that could fit requirements for risk assessment. The second article focuses on the quantitative assessment of the risks, impacts and costs. The overall objective is to carry out a cost-benefit analysis of the exposure control program that has been implemented in Guadeloupe since 2003, i.e. compare its cost with the benefit of reduced health impacts. Since the IOM report in 1981 [22] it is widely accepted that the cost of environment-related health effects should be consistently evaluated for decision making in public health.

## Methods

### Document search

In April 2013, we searched publications on pharmacokinetics, mechanisms of action and toxicity of chlordecone in MEDLINE and TOXLINE with the following key words: “Chlordecone” OR “Kepone” OR “143-50-0” without any other limitation. Some French scientific revues that are not indexed in MEDLINE were also screened. We excluded articles that are:

1. Abstracts only or conference proceedings
2. Editorials
3. Review articles on general topics (pesticides, organochlorides, reproductive toxins, etc.)
4. Anonymous references (no author name)
5. Reference duplicates
6. Eco-toxicity studies
7. The development of chemical or biochemical tests studies
8. Studies only of mirex, photomirex or kelevan

Based on information in the title and abstract, studies were classified into categories of interest for health risk assessment: Pharmacokinetics (absorption, distribution, metabolism, excretion); Mechanisms of action; Toxic Effects; Epidemiological Studies; Reviews and various.

### Chronic low dose hazard identification

We use 2 definitions of “low dose”. When analyzing mode of action (MOA) for key-event identification in mechanistic studies, the “low dose” hazard is: an adverse biological change occurring in the range of typical human exposures or at doses lower than what has been used in standard toxicology testing protocols [23]. Mechanistic studies that explore the key event for a MOA are not chronic and mostly not low dose, because one needs dose levels high enough to produce the effect. Our objective is to assess risk from chronic exposure to low dose of chlordecone, because this is the situation in Guadeloupe. The gap between the available knowledge and the information needs for decision making is very frequent in environmental health. Here, to decide if an adverse effect can result from low dose exposure the available knowledge comes from acute exposure studies and there is no alternative. First we identify the key-events of a MOA in mechanistic studies, and then if the data meet our criteria (see below) we assume the effect resulting from the key-events to be possible at chronic low-dose.

The first human data on adverse effects of chlordecone come from a cohort of workers poisoned in a chlordecone factory because of poor industrial hygiene. Exposures were very high (above 1 mg/l_blood_) and caused poisoning cases ranging in severity from a mild illness to a severe, totally disabling infirmity [24, 25]. Then toxicologists tried to understand the adverse effects observed among the workers. Hence, chlordecone has undergone many toxicological studies in vitro or in vivo using mostly relatively high doses (>10 mg/kg/d). These studies are old and almost entirely devoted to acute and subchronic effects. However, many mechanistic studies have identified the role of chlordecone in endocrine disruption, liver enzyme induction and unbalanced cellular energy. The analysis of available knowledge on the mode of action (MOA) aims to identify the effects of chlordecone that can occur at low doses, effects for which we will derive ERFs. After verifying consistency between the human and animal data or consistency between the animal species tested, the effects whose MOA meets at least 2 of the following 3 criteria will be selected as potentially effective at low dose:

1. The key-event for a MOA is observed at concentration ≤ 1 μM or dose < 1 mg/kg/d
2. The MOA is identical to that of a frequent human disease
3. A ubiquitous chemical follows the same MOA

For chlordecone we have chosen the low dose values based on an inventory of the range of doses used in toxicological studies. Most of them are between 10 and 40 mg/kg/d, for acute effect testing. As mechanistic studies are often based on one dosage, we consider that less than 1 mg/kg/d is a low dose for acute chlordecone exposure. The corresponding concentration is approximatively 0.1 μM based on information reported by End et al. 1979 [26]. They wrote that a brain concentration of 2 μM corresponded to an external dose of 40 mg/kg. This is a 0.05 μM per mg/kg that we rounded to 0.1 μM taking into account the fact that the brain is not the most impacted organ after a 40 mg/kg dose (see Figure I in Additional file 1).

### Deriving ERFs

Having identified via MOA analysis the possible effects at low dose, we derive the ERFs based on quantitative data from either human or animal studies. Studies used to derive an ERF must meet all the following criteria:

1. Epidemiological studies (cohort or case–control only); or animal studies following the OECD or US-EPA guidelines, with at least 2 dose groups in addition to the control group;
2. Study outcomes corresponding to one of the effects identified as occurring at low dose;
3. Quantitative measurement of chlordecone exposure (biomarkers or external doses);
4. Exposure duration ≥ 1 year or exposure in utero + post natal;
5. Medical or histopathological diagnosis of the disease;
6. Statistical tests of risk indicators;
7. Cofactor control in epidemiological studies (smoking, weight, age, gender, income level, other exposures, etc.).

Our study limited the ERF derivation to chronic exposure studies. In animal studies, this criterion corresponds to a length of at least 1 year of day to day consecutive exposure. Developmental studies need other criteria because in rodents developmental period is less than 1 year. Exposure only in utero or only post-natal will not adequately reflect the exposure of a new born and infant. It is why we selected only studies where exposures happen during gestation and early stage of life (postnal).

### ERF Derivation methods

In regulatory risk assessment, the objective is to protect the population from exposures that can lead to unacceptable risks. Here, we seek to know what are the actual risks in the French Indies population exposed to chlordecone. Accordingly, we are interested not only in the critical effect (generally defined as the first one to appear in the dose range tested) but all effects at low dose. There will be as many ERFs as effects of chlordecone at low dose for which there exist quantitative human or animal data. Then we are looking for representative average data and not just the data about the most sensitive specie or sex. If an effect is significant in both sexes or in both species, the ERF will be calculated as an average risk of both sexes or both species. However, if the effect is significant in only one of the 2 sexes, the ERF will be derived only from data of that sex and will apply in the risk assessment to persons of the corresponding sex. From animal studies as well as from epidemiological studies the derivation of ERFs involves 2 steps: choose a point of departure (POD) then draw a straight line from the POD to the origin [27].

### ERFs based on epidemiological studies

When deriving ERFs from epidemiological results, attention must be paid to causality [28]. Greenland indicated that, even with a systematic analysis of the well-known Hill’s criteria [29], causality will have little support or plausibility if the mode of action is unknown. As our framework starts with a systematic analysis of MOA, ERFs based on epidemiological will only be established for effects with known MOA.

Some epidemiological studies give an ERF directly. Otherwise, the ERF is estimated with the data presented in the study. The POD for the ERF is the risk indicator: relative risk (RR) or odds ratios. Extrapolation of this POD towards the origin is to divide the difference in risk (RR-1) by the exposure difference between the exposed group and the reference group. If an epidemiological study gives risk indicators for more than 2 groups (exposed/not exposed), use the risk indicator of the first group statistically different from the reference group. This rule is made to prevent obvious uncertainty propagation and is discussed with results on prostate cancer. For cancer effects, we assume that the probability of response is proportional to the duration of the exposure. In this case, the average (or median if average is not available) length of exposure in the study is used to standardize the ERF for a standard human lifetime (i.e. 70 years). The ERF is calculated with the following equation:

1 $$ \mathbf{E}\mathbf{R}\mathbf{F} = \left(\mathbf{R}{\mathbf{R}}_{\mathbf{a}} - \mathbf{1}\right)\ /\ \mathbf{a} \times TW $$

ERF = Exposure-response function (lifetime) expressed as invers of unit exposure

a = exposure difference between the reference group and the RR exposed group (in μg/L)

RR_a_ = relative risk related to the exposure difference “a”

TW = time weighted factor = standard human lifetime (70y)/mean duration exposure in the study (used only for cancer effects).

### ERF from animal studies

Experimental animal data are modeled as benchmark dose (BMD) [30]. A BMD is a dose corresponding to a specified probability of occurrence (the risk) of the adverse effect (the hazard), also called benchmark response (BMR). Many models (or functions) can be fit to toxicological data. The US-EPA has developed software that simultaneously estimates several models from study data. We use the BMDS v2.4.0 software for BMD determination from animal data. This software is freely available on the website of the US-EPA [31].

From animal data, the POD will be the best BMD. The best BMD is the one from the model with the best fit to the data, or, when statistical tests cannot decide between different models, the geometric mean of the respective BMDs. US EPA, in order to protect the population, uses the lower limit of the confidence interval at 95 % around the BMD (Noted BMDL) as POD. In our work the objective is to obtain the best estimate of the impacts and costs, so we have to think in terms of expected value and not in terms of safety margin. Therefore, we will use the central value of the BMD.

### Statistical tests before modeling the data

Original data from animal studies are organized into a data set which consists of 3 items: administered doses (e.g.: 0, 5, 10, 25, 50 mg/kg/d); number of animals per dose group; number of sick animals in each group. A data set is defined by gender, species and effect. Each data set is subjected to statistical tests before modeling. The first test, the Cochran Armitage trend test, verifies that the data set shows a significant trend of increasing incidence with increasing doses. It is performed with BMDS2.4.0 software. Second, the incidence rates of each dose group are tested against the incidence in the control group. The evaluation of the *χ*^2^ test is performed with the R software [32]. If the unilateral trend test (incidence should increase with dose) does not reject the null hypothesis (*p* > 0.025 or Z <1.96), there is no significant increased trend and the data set is excluded. A dose group not significantly different from the control group is excluded from modeling. After exclusion of one or more dose groups, the data set is modeled if there remain at least 2 dose groups other than the reference group, otherwise it is excluded.

### Benchmark dose modeling (BMD)

Briefly, in the BMDS2.4.0 software the models available for a dichotomous endpoint (quantal endpoint) are: Gamma*, Logistic, Log-Logistic*, Log-Probit*, Multistage*, Multistage-cancer, Probit, Weibull* Quantal-linear. The models marked with a star can be constrained by either power ≥ 1 (Gamma and Weibull) or slope ≥ 1 (Log-Logistic and log-probit) or number of polynomial coefficient ≥ 0 (Multistage). The Multistage-cancer model is available with 1, 2 or 3 stages. The polynomial coefficients of the Multistage-cancer model are always constrained to be ≥ 0. Thus, they are 16 models available. We do not give more details on the equations and parameters of these models because they are fully described in the user guide of the BMDS2.4.0 software [31].

BMDs can be computed relative to the background incidence as a Relative Risk (noted “extra risk” in BMDS software) or independent of the background incidence as an Absolute Risk (noted “added risk” in BMDS software). But since the background incidence in animals cannot be considered representative for humans, the BMDs will be computed as Absolute Risk, so that ERF can be used in any human population without knowing the background incidence. Each data set will be computed with the 16 models, and the best is selected according to the statistical tests described above.

### Choice of risk level (BMR)

As a general rule, the BMR (the probability of an adverse effect) should be selected close to the incidence in the first dose group so that modeling does not exceed too much the range of observations. A default 10 % BMR is recommended by US EPA for conventional animal data, because usually that corresponds to the power of studies with 20–50 animals per group. Here, we will use in all cases a 10 % BMR because this will facilitate comparaison with other BMDs derived by Public Agencies.

### Dosimetric adjustments

After modeling, animal BMDs are converted to human BMD (BMD_HED_) with a dosimetric adjustment (DAF), following the recommended use of “Body Weight^3/4^” as the default method [33]:

2 $$ \mathbf{B}\mathbf{M}{\mathbf{D}}_{\mathbf{HED}} = \mathbf{B}\mathbf{M}\mathbf{D} \times \mathbf{D}\mathbf{A}\mathbf{F} $$

DAF : Dosimetric Adjustment Factor for oral exposure (−) = (BW_A_/BW_H_)^1/4^

BW_A_ = Animal body weight (kg)

PC_H_ = human body weight (kg)

### Point of departure (POD)

The point of departure is the best BMD_10-HED_ (BMD_HED_ for a 10 % BMR). Out of the 16 model results, we exclude results that are significantly different from the data set: Goodness of Fit test with *p <*0.1; residual value near the BMR is higher than │2│; those that give aberrant BMD (usually infinitesimal); those for which the calculation of statistical tests failed; and those which have more parameters than the number of dose groups modeled. In quantal models, often a background parameter quantifies the probability that the outcome being modeled can occur in the absence of exposure. This information comes from the zero dose groups, so these groups are included in the number of groups taken into account. If several models remain acceptable after this procedure, a further test is applied: if the ratio between the lowest and the highest BMD is less than or equal to 3, then the POD is equal to the geometric mean of acceptable BMDs; if the ratio is greater than 3, then the POD is equal to the BMD having the smallest value of AIC (Akaike's Information Criterion). The AIC test refers to the principle of parsimony: the smallest value of AIC means, in a group of models, the one that best fits with the least possible parameters.

### Exposure-response function derivation

The ERF is the risk (here the BMR) divided by the point of departure (here the BMD_10-HED_):

3 $$ \mathbf{E}\mathbf{R}\mathbf{F} = \mathbf{B}\mathbf{M}{\mathbf{R}}_{10}/\mathbf{POD} $$

ERF = Exposure-response function expressed as (mg/kg/d)^−1^.

BMR_10_ = Benchmark response of 10 % (−)

POD = BMD_10-HED_ = Benchmark dose human equivalent for a BMR of 10 % (mg/kg/d)

Assuming that the probability of cancer at a constant dose increases linearly with exposure duration, a time weighting is applied to ERFs for carcinogenic effects if the exposure duration in the original study is not equal to the standard default lifespan of the animal species tested. Standard lifespans for humans, rats and mice, are respectively 70 years, 2 years and 2 years [34]. The weighting is to multiply the ERF by the ratio of the standard lifespan and the duration of exposure in the study.

### Grouping data sets

Because grouping data sets can strengthen the statistical power and robustness of the derived BMD it could be interesting to group data sets before modeling them if they meet some statistical and biological criteria. Unfortunately there was no possibility for grouping data sets with the available data on chlordecone. So this point will not be detailed here. Interested readers can find description of the methods and discussions on advantage and weakness elsewhere [35].

### Exposure data from the Guadeloupe population

Two approaches have been used in the past to assess exposures to chlordecone in the Guadeloupe population: biomarker measurement (called here “blood chlordecone”) and exposure estimated by food consumption combined with concentrations of chlordecone in food (called here “dose by ingestion”). An ad hoc study published in 2010 found that dose by ingestion in Guadeloupe is poorly correlated (R^2^ = 0.20) with blood chlordecone [20]. Moreover, for the comparative analysis of health costs and expenditures for preventive actions (see article 2) we need data spaced over time to estimate the exposure difference attributable to the exposure reduction program. The chronology of the public actions for reducing exposure was reported in a recent study [36]. Accordingly, one can consider that the exposures measured until 2003 describe the situation before the reduction program. Those measured since 2004 are influenced by this program [1–3].

Due to the specific persistence of chlordecone in blood, the best indicator of chronic exposure appears to be the blood chlordecone concentration (expressed in μg/L_blood_). Furthermore there are no ingestion exposure data before 2005, which prevents estimating the ingestion exposure difference due to the program. Therefore we sought all studies that have measured blood chlordecone concentrations in Guadeloupe.

### Substitution method for censored data

Where measurements are below the limit of detection (LD), the maximum likelihood estimation method (MLE) is often considered the gold standard for substituting values for the censored data. Ganser and Hewett published a simplified method called “β-substitution” giving very similar results, sometimes better than the MLE method and always better than the simplified DL/2 or DL/√2 methods [37]. Here, the mean exposure of groups that are below the LD will be estimated by using the β-substitution method from Ganser and Hewett.

### Data preparation

Distributions that are not normal can be described by the value of quartiles or other percentiles deemed appropriate. With quartiles, the population is divided into 4 groups: those whose exposure is between zero and the 25th percentile (P25), then those between P25 and P50, those between P50 and P75 and finally those from P75 to the maximum value. When a sample includes censored data due to the LD, the first group is divided in 2 parts: those whose exposures are somewhere between 0 and LD and those between LD and P25. For this particular group average value will be calculated according to β-substitution method called “βM”. Finally, the average exposure in each group shall be calculated as follows:

1st group (results < LD), exposure equals the mean β-substitution (βM)

2nd group (from βM to P25), exposure equals the geometric mean of βM and P25

3rd group (P25-P50), exposure equals the mean of P25 and P50

4th group (P50-P75), exposure equals the mean of P50 and P75

5th group (P75-max), exposure equals the geometric mean of P75 and max.

If the proportion of results < LD exceeds 25 %, then the second group takes the average value of βM and P50 and there are only 4 exposure groups. If the proportion of results < LD exceeds 50 %, then the second group takes the average value of βM and P75 and there are only 3 exposure groups.

## Results

Available studies on the toxicity of chlordecone for low dose hazard identification (mode of action and key-events) are numerous but generally old. These studies were analyzed in depth, and the details are reported elsewhere [35]. Here we provide a summary of the main results for MOA and key events. The studies were mainly focused on the acute toxic effects observed in workers poisoned in the 70s because of poor industrial hygiene in a chlordecone factory (Hopewell, USA) [24, 25, 38–41].

Chlordecone is a known endocrine disruptor in humans with low agonist affinity for estrogen receptor ER-α [42–49]. There are numerous studies on estrogen affinity in other species. They are less relevant and not different from those in humans. However, its adverse effects involve mechanisms partially comparable to estradiol. Chlordecone follows other toxicological pathways, mainly:

- induction of hepatic cytochrome [50–69].
- inhibition of ATPase in the liver [52–55, 69].
- inhibition of ATPase in the brain [70–82].
- perturbation of neurotransmitters such as dopamine, epinephrine, norepinephrine, serotonin, beta-endorphin, enkephalin and gamma-aminobutyric acid [50, 83–100].
- leakage of intracellular calcium [26, 101–106].

The US EPA and the IARC considered chlordecone non-genotoxic. Some epigenetic mechanisms of chlordecone carcinogenicity have been tested on human cell culture. It has proved capability to: inhibit arginine methylation [107], inhibit aromatase activity [108], cause DNA single strand breaks [109], limit intercellular communication [110, 111] and increase the activity of certain protein kinases (MAPK, PI3-K) [112]. Most of these effects are also observed in laboratory animals [113–116]. Finally, a recent study showed the ability of chlordecone to promote angiogenesis and neovascularization via activation of ER-α at low doses [117].

Our in depth analysis of MOA and key-events led us to consider 4 non-genotoxic effects of chlordecone potentially effective at low dose:

- increased mostly hormone-dependent cancers (prostate and breast),
- hepatotoxicity,
- neurotoxicity,
- and developmental impairment (brain and probably heart),

For cancer promotion, the MOA effective at low doses are aromatase inhibition [108], disruption of intercellular communication [116, 118], and the increase of angiogenesis [117]. The ubiquitous substances that share the same MOA are bisphenol A, DDT, DDE and lindane. Regarding hepatotoxicity, the MOA is the induction of cytochrome P450 [60, 62], which can also led to cancer development via reactive oxygen species production. Induction of CYP450 is a reversible effect but it is shared by numerous chemicals ubiquitous in the human environment: 2,3,7,8-TCDD, PCBs and lead. Regarding neurotoxicity in adulthood, the key-event observed at low doses is a decrease of ATPase activity [73, 76, 80, 81]. This can be related to human diseases, for example lower brain ATPase activity in the elderly may contribute to the onset or worsening of neurodegenerative diseases such as Parkinson’s disease or Alzheimer’s. This MOA is shared by acrylamide and cadmium and other ubiquitous metals. Other key-events were studied for neurotoxicity in adulthood, like cellular calcium leakage or perturbation of neurotransmitters (see references above) but unfortunately not studied at low doses. Moreover, we made the hypothesis that if those key-events (decreased ATPase cellular, calcium leakage and some specific endocrine disruption) occurred in utero and during early months of life, the central nervous system development could be impacted leading to cognitive impairment. These key-events have not been studied in “developmental studies”. In vivo developmental studies involved in low dose neurotoxicity looked mostly at altered stress response [119–122] that is less relevant in human. Our hypothesis is reinforced with the fact that other developmental neurotoxic substances (i.e. heavy metals) that impair cognitive ability of children, share some of the same cellular key-events. At least, authors of the TIMOUN cohort study (description of this study appears in next section) have postulated the same hypothesis, mostly based on endocrine disruption, and found inverse correlation between chlordecone blood concentration of mothers during pregnancy and decrease in neurological performance in boys at 18 months of age but not in girls (this discrepancy could confirm that an endocrine mechanism is involved).

Nephrotoxicity is not among possible low dose effects according to our analysis of the MOA, mainly because the underlying mechanisms remain unclear (Is the chlordecone responsible for triggering autoimmune diseases that will subsequently impact the kidneys? Or it has a direct toxicity to the kidney glomeruli?), there are inconsistencies between humans and animals and inconsistencies between different rodent species or strains. However, a doubt remains as to the role of chlordecone in promoting autoimmune diseases like systemic lupus erythematosus. Kidney damage is the critical effect chosen by several health safety agencies [17, 18, 123]. All have derived a reference dose from data of the Larson et al. study [7]. In order to compare the risk assessment of this effect with others, we will derive an ERF for kidney damages following the same methods.

For deriving ERFs we found quantitative studies (epidemiological and toxicological) for only 3 out of the 4 possible low-dose effects of chlordecone: cancer promotion, liver toxicity, and developmental impairment. Neurotoxicity in adults is a possible low dose effect of chlordecone but for which no ERF can be derived up to now (December 2015) for lack of a quantitative study.

In humans, studies of workers poisoned in the Hopewell factory are not suitable for deriving chronic ERFs because the exposure time could have been less than one year; moreover, all neurological, hepatic, or fertility symptoms disappeared some weeks after exposure cessation [24, 25, 38–41]. There are 2 recent epidemiological studies in the French Antilles with exposure time longer than 1 year and eligible effects. The first is a case control study that found a statistically significant dose-dependent association between risk of prostate cancer and blood levels of chlordecone [11]. The second is a cohort of mothers and children followed from birth (cohort TIMOUN). Published in 2012, the first results have identified signs of neurotoxicity in children 7 months after birth correlated with chlordecone levels in cord blood and breast milk [12]. New results published in 2013 confirmed the previous results 18 months after birth and with more detail. Alteration of neurobehavioral development at 18 months is characterized by a decrease in fine motor skills statistically significant only in boys [13].

In animals 3 chronic (>1 year) and multi dose studies meet our criteria [7, 19, 124]. The NCI study shows a significant increase in liver cancers (carcinomas) in rats (Osborne-Mendel) and mice (B6C3F1) [19]. It is of intermediate quality because the initial doses, due to their excessive toxicity, were reduced during the course of the experiment. In addition, the control groups consisted of 10–20 animals against 50 in the dose groups. This weakness was offset by the addition of control animals from other experiments in the same laboratory during the same period. The doses were subject to time weighted averaging, which is acceptable from a mathematical point of view but maybe not from a biological point of view. The Reuber study [124] looked at liver cancers in albino rats (unspecified strain). Published data are incomplete and far from scientific quality standards. Nevertheless, the results could be modeled for comparison. The Larson et al. study [7] reports several effects including hepatotoxicity. Six dose groups were tested (1, 5, 10, 25, 50, 80 ppm), but in the last 2 groups all animals died during the first 6 months (unspecified cause of death). The dose group at 1 ppm is part of a separate experiment. There are also high rates of missing animals at the end of the study, ranging from 30 % in the controls to over 80 % in the group of 25 ppm. The causes of these “disappearances” are not reported. Despite these weaknesses, the study data could be modeled because its scientific qualities were considered sufficient to support a reference dose derivation by many health safety agencies [17, 18, 125].

Available data for exposure characterization come directly from epidemiological studies conducted in Guadeloupe since 1999. They provide some distributions of the blood chlordecone concentration in the sampled population. The first one, called INSERM, is a cross-sectional study held between 1999 and 2001 with 2 different sample populations: agricultural workers (*n =* 42) and non-agricultural workers (*n =* 45). It aimed to measure the exposure of men to chlordecone and to study its impact on male fertility [15]. We will use only non-agricultural worker data because the exposures of the agriculture workers were much too high to be representative of the general population. The second study, called HIBISCUS and conducted in 2003, aimed to investigate the cross sectional prevalence of exposure to chlordecone in pregnant women (*n =* 112) and neonates (*n =* 109) [126]. The third, called cohort TIMOUN and carried out between 2004 and 2007, aimed to assess the impact of the exposure on the course of pregnancy (*n =* 371) and on the development of the child (*n =* 265) [20]. The fourth, called KARUPROSTATE and carried out between 2004 and 2007, is a case–control study (*n =* 623/671). It aimed to investigate the relationship between the risk of prostate cancer and exposure to chlordecone in men aged over 44 years [11]. The quartiles of measured Cl-b levels in these studies were reported in a recent review article [127].

The ERFs derived from the available epidemiological or experimental studies are presented in Table 1. The benchmark dose model results are shown in Tables 3, 4 and 5.

**Table 1:** Exposure response function (ERF) and main information taken into account for derivation

Effect	Study	Design	Species/sex	Exposure	duration	DAF (−)	BMR or ΔRR	POD	TW (−)	Raw ERF_TW_	Absolute ERF_TW_	Unit*	Population affected	Dominant source of variability of risk estimated with this ERF
Prostate cancer	A	Case/control	Human/M	Digestive	33 y	na	0.77	6.741	2.12	0.242^#^	0.0019	(μg/L_blood_)^−1^	Men > 45 years	IC_95 %_ OR
Developmental cognitive impairment	B	Prospective cohort	Human/M	in utero + post-natal	Gestation + post-natal	Slope factor taken from the study				−0.320	−0.320	(μg/L_cord-blood_)^−1^	Male newborn	Equivalence between 1 QI point and 1 fine motor point at 18 months of age
Liver cancer	C	Experimental	Mouse/FM	Digestive	80 wk.	0.15	0.10	0.048	1.30	2.692	2.692	(mg/kg/d)^−1^	All	Conversion of ingestion dose to blood concentration
Renal lesions	D	Experimental	Rat/F	Digestive	20 month	0.26	0.10	0.012	na	7.923^#^	0.0022	(mg/kg/d)^−1^	Women	Conversion of ingestion dose to blood concentration

A: Multigner 2010 [11], B: Boucher 2013 [13]; C: NCI 1976 [19]; D: Larson 1979 [7]. DAF: dosimetric adjustment factor for animal to human dose conversion. BMR: benchmark response. ΔRR = RR-1. POD = BMD_10_-_HED_ or Δexpo. BMD_10_-_HED_ = BMD_10_ × DAF. Δexpo = average exposure in RR group less average exposure in referent group. TW: time weighted factor (only for cancer effects). ERF: Exposure response function. Raw ERF_TW_: ERF resulting from Eq. 1 or 3 and weighted for time if necessary: Raw ERF_TW_ = BMR/POD × TWF. Absolute ERF is the raw ERF from which background incidence (I_0_) was subtracted if necessary (POD derived from relative risk or effect restricted to a part of the population): prostate cancer in men > 45y (I_0_ = 0.0079), renal lesions (I_0_ = 0.00027). For renal lesions, the background incidence is annual new cases of women with erythematous systemic lupus. Absolute ERF_TW_ = Raw ERF_TW_ × I_0_. SLE: systemic lupus erythematosus. F = Female. M = male. “na” not appropriate

* Same unit for raw and absolute ERF. The unit of the POD is the inverse of the ERF unit. Cancer ERFs are for lifetime exposure

# Those ERFs must be used together with background incidence rates. For renal lesions, the incidence is for women with erythematous systemic lupus

**Fig. 1:**
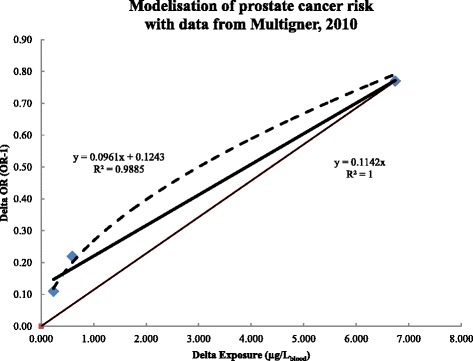
Linear regression for the ERF from three exposure groups in the KARUPROSTE study. ERFs calculated with equation 1 for each exposure group in the KARUPROSTATE study are plotted as squares. Dashed line is the “best” regression (best R^2^) between delta exposure and delta risk (Power function: y = 0.2685x^0.5664^, R^2^ = 0.9919). The thick line is the linear regression (equation and R^2^ values shown to the left). The thin line is the graphical representation of the final ERF (equation and R^2^ values shown to the right) derived from the first exposure group that significantly differs from the reference group, i.e. the highest exposed group

For prostate cancer, the KARUPROSTATE study includes 623 cases (new cases of prostate cancer diagnosed in Guadeloupe between 2004 and 2007) and 671 controls. Exposures are measured by Cl-b (LD = 0.25 μg/L_blood_). The authors found an increased risk of cancer significantly correlated with the increase in current Cl-b (p trend = 0.002). The odds ratio is only significant in the most highly exposed group. The Table 2 presents the ERFs of the 3 exposure groups, calculated using Equation 1. The ERF retained is that of the most exposed group: 0.114 (μg/L_blood_)^−1^. Linear regression of the 3 ERFs gives a fairly similar slope factor of: 0.096 (μg/L_blood_)^−1^ (cf. Fig. 1). The mean exposure duration was 33y, thus the ERF weighted for a standard lifespan exposure is 0.242 (μg/L_blood_)^−1^. According to the cancer registry, the background incidence of prostate cancer in Guadeloupe is 259 per 100 000 (gross rates, all ages), based on an average 510 new cases a year [128]. Assuming there is no risk of prostate cancer before the age of 44 years and taking the number of men aged over 44 years (65000 in 2007, according to INSEE), the incidence rate is 770 per 100000 (510 cases/65000 men). Hence, the ERF based on relative risk can be converted to an absolute risk equivalent of 0.0019 (μg/L)^−1^ (=0.242 (μg/L)^−1^ × 0.0077). The latter applies to any population of men aged over 44 years.

**Table 2:** Prostate cancer risk increase and ERF calculation (data from Multigner, 2010 [11])

Exposure groups in the study (μg/L_blood_)	OR_multivar_ (IC_95 %_) (−)	Delta OR (−)	Averaged exposure (μg/L_blood_)	Delta Expo (μg/L_blood_)	ERF (μg/L_blood_)^−1^
<0.25 (LD)	1	Ref.	0.125^a^	Ref.	Ref.
>0.25 to 0.47	1.11 (0.75-1.65)	0.11	0.360^b^	0.235	0.468
>0.47 to 0.96	1.22 (0.82-1.83)	0.22	0.716^b^	0.591	0.373
>0.96*	**1.77 (1.21-2.58)**	**0.77**	**6.866** ^**c**^	**6.741**	**0.114**

ERF are calculated with Eq. 1

Results in bold face are used to derive the ERF

LD = limit of detection. Ref. = reference group

Delta OR = OR - 1; delta expo = average exposure value – 0.125; ERF = delta OR/delta expo

* The maximal value in the study is 49.1 μg/L (cf. Guldner 2011 [127])

a = Value stated by Multignier et al. to compute the test for trend

b = arithmetic mean of the minimum and maximum values of this exposure group

c = geometric mean of the minimum and maximum values of this exposure group, respectively: 0.96 μg/L and 49.1 μg/L

For developmental impairment, in the prospective cohort TIMOUN cognitive ability scores were measured in 141 young children exposed to chlordecone during pregnancy and lactation. The exposures were measured at birth by chlordecone concentration in cord blood (μg/L_cord-blood_) and 3 months later by the chlordecone concentration in breast milk. The latter is not used here. There are 30 % of censored data in cord blood measurements (LD = 0.06 μg/L_cord-blood_). Cognitive abilities were measured at age 7 months [12] then at 18 months [13]. At 18 months, the authors used a standardized procedure to evaluate cognitive development named “Age and Stage Questionnaire” (ASQ). It includes 5 items: “personal-social”, “Communication”, “Problem solving”, “Fine motor skills”, and “Gross motor skills”. Only fine motor skills scores of boys were significantly and inversely correlated with the concentration of chlordecone in the cord blood. According to the authors, this is consistent with the available knowledge including the endocrine disruption mechanism. This article gives a slope coefficient of the multivariate linear regression of −0.32 points of fine motor skills per μg_chlordecone_/L_cord-blood_. The relationship between the ASQ scores and IQ scores is not well known. For the purposes of our study we considered that an ASQ fine motor point equals an IQ point. According to our hypothesis the ERF would be −0.32 IQ point per μg/L_cord-blood_ and applies only to newborn males.

The ERF for liver cancer is based on animal studies. With the NCI data on hepatic carcinomas, we obtained 4 sets of data: female and male rats, female and male mice. There is no significant difference between the control group and the first dose group in male and female rats (see: Additional file 2: Table B). After excluding the first group in both datasets there remains only one dose group. This is insufficient for modeling, so those datasets are excluded. In B6C3F1 mice, all dose groups were significantly different from the control group and trend tests are significant. The grouping of males and females before modeling is not recommended because the incidence of hepatocellular carcinomas in the female control group is zero while it is 21 % in males (*p <*0.0006). Only one out of the 16 tested models fits the female data (unconstrained Multistage). Ten models differ significantly from the data (*p <*0.1). The results of Probit and Logistic models exhibit residuals greater than 2. Computation failed with 2 models: log-Probit, log-logistic. One model gives an outlier result: gamma (see Table 3). The BMD_10_ of the Multistage model is 0.274 mg/kg/day, and a BMD_10-HED_ of 0.0412 mg/kg/d is calculated with a DAF of 0.15 (see DAF calculation in Additional file 2: Table A).

**Table 3:** BMD_10_ for liver carcinomas in female mice (from NCI, 1976)

Model	Total number of parameters in the model	AIC	Goodness of fit test P-value	Scaled residual near BMR	BMD_10_ (mg/kg/d)	BMD_10-HED_ (mg/kg/d)
Gamma-Restricted	3	147.182	0.014	0	0.671	0.1005
Gamma	3	141.305	0.568	0	2E-35	na
Logistic	2	166.361	0.000	3.47	1.824	0.2734
LogLogistic-Restricted	3	143.927	0.081	0	0.464	0.0695
LogLogistic	3	139.233	0.881	0	computation failed	
LogProbit-Restricted	3	149.575	0.004	0	1.149	0.1722
LogProbit	3	139.233	0.881	0	computation failed	
Multistage-Restricted	2	147.182	0.014	0	0.671	0.1005
Multistage	**2**	**140.979**	**1.000**	**0**	**0.275**	**0.0412**
Multistage-Cancer 1	2	147.182	0.014	0	0.671	0.1005
Multistage-Cancer 2	3	147.182	0.014	0	0.671	0.1005
Multistage-Cancer 3	4	147.182	0.014	0	0.671	0.1005
Probit	2	164.975	0.000	3.509	1.704	0.2553
Weibull-Restricted	3	147.182	0.014	0	0.671	0.1005
Weibull	3	147.182	0.014	0	0.671	0.1005
Quantal-Linear	3	147.182	0.014	0	0.671	0.1005
Minimal BMD						na
Maximal BMD						na
Ratio max/min						na
BMD from best AIC model						**0.0412**
Geometric mean of BMDs for acceptable models						na

The benchmark response is 10 %. AIC: Akaïke’s Information Criterion. BMD_10_: Benchmark dose for 10 % excess risk. Results in bold are acceptable models according to these criteria: total number of parameters does not exceed the number of groups modeled (*n =* 3); and *p*-value > 0.1; and scaled residual < │2│ and no computation error or unrealistic graphical BMD_10_. If ratio max/min is less than 3, then geometric mean of acceptable models is selected, else the best AIC model is selected. “na”: not applicable

In male mice, 4 models fit the data (Logistic, Multistage-cancer1, Probit, quantal-linear). Goodness of fit test was not computable with the other models (see Table 4). The ratio between the smallest and the largest BMD is 2.1. The geometric mean BMD_10_ is equal to 0.372 mg/kg/d and the BMD_10-HED_ equal to 0.057 mg/kg/d (DAF = 0.152). The geometric mean of the 2 BMD_10-HED_ (male mice and female mice) is 0.0483 mg/kg/d, and the ERF is 2.071 (mg/kg/d)^−1^ for an absolute risk of 10 %. In this study, mice were exposed for 80 consecutive weeks, shorter than the standard lifespan. A temporal weighting of 1.30 (=104/80) must therefore be applied. The ERF for liver cancer with a lifetime exposure in men and women is 2.692 (mg/kg/d)^−1^.

**Table 4:** BMD_10_ for liver carcinomas in male mice (data from NCI, 1976)

Model	Total number of parameters in the model	AIC	Goodness of fit test *P*-value	Scaled residual near BMR	BMD_10_ (mg/kg/d)	BMD_10-HED_ (mg/kg/d)
Gamma-Restricted	3	157.911	NA	0	0.841	0.1279
Gamma	3	157.911	NA	0	0.841	0.1279
Logistic	**2**	**155.911**	**0.9773**	**0.002**	**0.545**	**0.0828**
LogLogistic-Restricted	3	157.911	NA	0	1.136	0.1725
LogLogistic	3	157.911	NA	0	1.136	0.1725
LogProbit-Restricted	3	157.911	NA	0	1.078	0.1638
LogProbit	3	157.911	NA	0	1.078	0.1638
Multistage-Restricted	2	157.911	NA	0	0.536	0.0814
Multistage	**2**	157.911	NA	0	0.536	0.0814
Multistage-Cancer 1	**2**	**156.027**	**0.733**	**0.007**	**0.257**	**0.0391**
Multistage-Cancer 2	3	157.911	NA	0	0.536	0.0814
Multistage-Cancer 3	4	159.911	NA	0	0.373	0.0567
Probit	**2**	**155.914**	**0.9557**	**−0.005**	**0.535**	**0.0812**
Weibull-Restricted	3	157.911	NA	0	0.674	0.1024
Weibull	3	157.911	NA	0	0.674	0.1024
Quantal-Linear	**3**	**156.027**	**0.733**	**0.007**	**0.257**	**0.0391**
Minimal BMD						0.0391
Maximal BMD						0.0828
Ratio max/min						2.1
BMD from best AIC model						0.083
Geometric mean of acceptable models BMD						**0.057**

The benchmark response is 10 %. AIC: Akaïke’s Information Criterion. BMD_10_: Benchmark dose for 10 % excess risk. Results in bold are acceptable models according to these criteria: total number of parameters does not exceed the number of groups modeled (*n =* 3); and p-value > 0.1; and scaled residual < │2│ and no computation error or unrealistic graphical BMD_10_. If ratio max/min is less than 3, then geometric mean of acceptable models is selected, else best AIC model is selected. “NA”: not applicable

The Reuber study provides 2 sets of data for liver cancer (male and female rats). None of the 3 dose groups were significantly different from the control group (see: Additional file 2: Table C). According to the criteria exposed in the section “statistical test before modeling”, the results of this study cannot be modeled (no significant difference from the control group).

In the Larson study, examination of rats showed some hyperplasia lesions (3 females at 10 ppm and 1 female and 2 males at 25 ppm) that could be precancerous (without histopathological confirmation). The doses are expressed as ppm chlordecone in the diet. From the average food consumption (in g_food_/kg/d) by group and by sex measured at different time intervals (5, 13, 26, 52 and 104 weeks) we calculated the weighted average doses of males and females with this data (see: Additional file 2: Table D). Only one dose group is significantly different from the control group in males (25 ppm) or in females (10 ppm) (see: Additional file 2: Table E). Hence, these datasets cannot be modeled (not enough of dose groups for modelisation).

For other endpoints of hepatotoxicity there is no data meeting our criteria. The Larson study shows hepatotoxicity characterized by some hyperplasia in the high dose group and changes of liver fat in all dose groups. Two data sets (male and female) were created by adding these 2 effects (see: Additional file 2: Table F). In males as in females only a single dose group is statistically different from that of the control group. These data sets are therefore excluded. There is no other quantitative data for chronic hepatotoxicity of chlordecone.

For renal dysfunction, ERF data are from animal studies. The Larson study has found a significant adverse effect of chlordecone on kidneys. In females the trend test is significant. The incidence of kidney damage (15 %) in the first dose group is not different from the control group (12 %) contrary to the other dose groups (see: Additional file 2: Table G). This dose group is excluded from modeling. In males, the trend test was not significant (*p =* 0.094). No dose group is different from the control group whose background incidence (55 %) is already very high. Combining the 2 sexes is not acceptable because of a significant difference in the incidence in the control groups of males and females (*p <*0.0005). Only the female data set, without the first dose group, can be modeled to estimate a BMD. All models, except Logistic and Probit, fit the data set (see Table 5). The ratio between the smallest and largest BMD was 29, so the BMD_10_ having the smallest AIC score (LogLogistic restricted) is retained: 0.0495 mg/kg/d. From this a BMD_10-HED_ of 0.0126 mg/kg/d (DAF = 0.255) is calculated, yielding an ERF of 7.923 (mg/kg/d)^−1^ for increased kidney damage among women with SLE (Systemic Lupus Erythematosus). The ERF is limited to women with SLE because glomerulosclerosis in mice and rats are found only in strain prone to SLE [10]. The incidence of SLE in women remains poorly quantified between 15 and 50 per 100 000. We assume a geometric mean incidence rate of 27 per 100 000 to convert the ERF for women with SLE into an ERF of 0.0022 (mg/kg/d)^−1^ (=7.923 (mg/kg/d)^−1^ × 0.00027) for all women.

**Table 5:** BMD_10_ for glomerulosclerosis in female rat (data from Larson, 1979, dose group 1 excluded)

Model	Total number of parameters in the model	AIC	Goodness of fit test *P*-value	Scaled residual near BMR	BMD_10_ (mg/kg/d)	BMD_10-HED_ (mg/kg/d)
Gamma-Restricted	**3**	**73.996**	**0.218**	**−0.25**	**0.0857**	**0.0219**
Gamma	**3**	**74.198**	**0.594**	**0.01**	**0.0043**	**0.0011**
Logistic	2	78.734	0.009	1.19	0.2102	0.0537
LogLogistic-Restricted	**3**	**72.206**	**0.848**	**−0.02**	**0.0495**	**0.0126**
LogLogistic	**3**	**74.083**	**0.679**	**0.01**	**0.0259**	**0.0066**
LogProbit-Restricted	**3**	**73.859**	**0.215**	**−0.15**	**0.1235**	**0.0315**
LogProbit	**3**	**74.093**	**0.671**	**0.01**	**0.0288**	**0.0073**
Multistage-Restricted	**2**	**73.996**	**0.218**	**−0.25**	**0.0857**	**0.0219**
Multistage	**2**	**73.915**	**0.969**	**0.00**	**0.0552**	**0.0141**
Multistage-Cancer 1	**2**	**73.996**	**0.218**	**−0.25**	**0.0857**	**0.0219**
Multistage-Cancer 2	**3**	**73.996**	**0.218**	**−0.25**	**0.0857**	**0.0219**
Multistage-Cancer 3	4	73.996	0.218	−0.25	0.0857	0.0219
Probit	2	79.168	0.016	1.28	0.2378	0.0607
Weibull-Restricted	**3**	**73.996**	**0.218**	**−0.25**	**0.0857**	**0.0219**
Weibull	**3**	**73.996**	**0.218**	**−0.25**	**0.0857**	**0.0219**
Quantal-Linear	**3**	**73.996**	**0.218**	**−0.25**	**0.0857**	**0.0219**
Minimal BMD						0.001
Maximal BMD						0.032
Ratio max/min						29
BMD from best AIC model						**0.0126**
Geometric mean of acceptable models BMD						Not applicable

The benchmark response is 10 %. AIC: Akaïke’s Information Criterion. BMD_10_: Benchmark dose for 10 % excess risk. Results in bold are acceptable models according to these criteria: total number of parameters does not exceed the number of groups modeled (*n =* 4); and p-value > 0.1; and scaled residual < │2│ and no computation error or unrealistic graphical BMD_10_. If ratio max/min is less than 3, then geometric mean of acceptable models is selected, else best AIC model is selected. “na”: not applicable

Exposure data are from epidemiological studies and presented in Table H of Additional file 2. Parameter values of the β-substitution model for each study are also in Additional file 2 (Table I). The average exposure values spread into 5 groups are shown in Table 6. To compare these exposure data with the RfD (0.5 μg/kg/d) we converted the RfD to a blood chlordecone concentration equivalent with a conversion factor CF_e/i_ of 0.0064 (μg/kg/d)/(μg/l) (see details in Additional file 1) giving a value of 7.81 μg/l. This level is reached only in the last group of exposure (group 5) of 2 populations: adult men and women only before 2003. The weighted mean (last column in Table 6) of all groups are below this value. Therefore, blood chlordecone concentrations measured in epidemiological studies are mainly under the RfD value. If chlordecone would not cause some effects at this low exposure levels, epidemiological studies that take cofactors and confounders into account would probably not be able to detect significant associations especially in newborns. The analytical limits of detection (LD) were lowered between the first studies (1.5 and 0.5 μg/L) and those realized after 2003 (0.25 μg/L). Despite this decrease, the rates of results below the LD increase significantly after 2003. This indicates that the exposure decrease is associated with a strong shift in the distribution to the left. However, the maximum values do not seem to follow the same decreasing trend and are even higher after 2004 in pregnant women and newborns. Because of the known kinetics of chlordecone in human blood (half-life of about 6 months), these observations could be interpreted as a decrease in exposures of continuous and homogeneous nature, for example via tap water, and the persistence of higher intermittent exposures, for example via food consumed occasionally. These exposure data will subsequently be used to assess the risks and impacts and the health and economic benefits attributable to the exposure control program.

**Table 6:** Blood chlordecone concentrations: averages by exposure group for purpose of risk assessment

Study name (time period)	Population	Group 1 (μg/L)	Group 2 (μg/L)	Group 3 (μg/L)	Group 4 (μg/L)	Group 5 (μg/L)	Weighted mean
INSERM (1999–2001)	Men 20-45y	1.06	1.56	3.90	7.25	14.79	7.14
KARUPROSTATE (2005–2006)	Men >44y	0.17	0.22	0.40	0.90	7.60	2.32
Difference : after 2003 - before 2003		−0.90	−1.34	−3.50	−6.35	−7.19	−4.82
HIBISCUS (2003)	Mother 17-45y	0.32	0.62	1.70	3.05	8.05	3.43
TIMOUN (2004–2007)	Mother 17-45y	0.18	nc	0.29	0.65	4.17	1.37
Difference : after 2003 - before 2003		−0.15	−0.40	−1.30	−2.15	−0.45	−1.11
HIBISCUS (2003)	Newborn	0.39	nc	0.55	0.95	2.11	1.00
TIMOUN (2004–2007)	Newborn	0.19	nc	nc	0.24	2.62	0.81
Difference : after 2003 - before 2003		−0.21			−0.71	+0.51	−019

Group 1 = βM value. βM is calculated with the β-substitution method from Ganser and Hewett 2010 [37]. Group 2 = geometric mean βM-P25. Group3 = arithmetic mean P25-P50. Group 4 = arithmetic mean P50-P75. Group 5 = geometric mean P75-Max. When a percentile value is censured by the limit of detection (example: P25 in Hibiscus newborn), means are not calculated. Then, for the next group the mean is calculated with βM and percentile value. Weighted means are the sum of group values weighted by the proportion of population in each group. Group 1 + group 2 = 25 %, group 3 = 25 %, group 4 = 25 % group 5 = 25 %. For TIMOUN new-born group 1 + 2 + 3 = 50 %, group 4 = 25 % and group 5 = 25 %

## Discussion

Our literature search has been updated from January 2014 up to March 2016. Only 5 new toxicological mechanistic studies were found. Two of them confirm estrogenicity of chlordecone and were more designed to develop new estrogenic tests [129, 130]. One study shows how chlordecone among others regulates the liver drug transporter activity and expression [131]; that may contribute to the liver toxicity that we have already taken into account. Another study [132] does not add anything beyond what is already known about chlordecone neurotoxicity. The last study, using quantitative PCR, revealed an induction of genes involved in defense mechanism against oxidative stress (catalase and selenium-dependent glutathione peroxidase) in prawns exposed to low environmental concentrations of chlordecone after 12 and 24 h of exposure. In prawns reared in a contaminated farm, transcription of genes involved in the biotransformation process (cytochrome P450 and glutathione-S-transferase (GST)) was induced after 8 days of exposure [133]. None of these studies change our conclusions on key-events and MOA.

Several new epidemiological studies have been published, mostly based on data from the TIMOUN cohort [134–138]. No significant associations were observed between chlordecone exposure and the risk of preeclampsia or gestational diabetes mellitus. This study suggests an inverse association between chlordecone exposure during pregnancy and gestational hypertension. The authors conclude that further studies are required to determine the underlying mechanism [134]. A 1-log10 increase in chlordecone concentration was associated with a decreased length of gestation (−0.27 weeks) and an increased risk of preterm birth (60 %). These associations may result from the estrogen-like and progestin-like properties of chlordecone [136]. Perinatal exposure to chlordecone may affect thyroid-stimulating hormone (TSH) and thyroid hormone levels at 3 months, differently according to the sex of the infant. This disruption however did not appear to intervene in the pathway between prenatal chlordecone exposure and fine motor development of children [137]. Chlordecone in cord blood was associated with a higher BMI in boys at 3 months and in girls at 8 and 18 months. Postnatal exposure was associated with lower height, weight and BMI at 3, 8 and 18 months, particularly in girls. Chlordecone exposure may affect growth trajectories in children aged 0 to 18 months [138]. Based on incident prostate cancer cases from 1981 to 2005 in Martinique Cancer Registry, incidence was found to increase by 5.07 % annually during this period of time. But an inverse association between exposure area and prostate cancer risk was found, with the highest prostate cancer incidence observed in urban zones showing the lowest soil contamination levels by chlordecone [135]. The studies confirm and amplify previous knowledge on developmental effects of chlordecone. The last study could raise doubts about chlordecone and prostate cancer. Nevertheless, the strength of an ecological design is not enough to negate the results of a case control designed study (here the KARUPROSTATE study). Moreover, because exposure to chlordecone is mainly due to food contamination (vegetable and seafood product) exposure to chlordecone is not limited to people living on contaminated soil. No ERF can be derived from these new epidemiological studies.

Following NRC 2009 recommendations for health risk assessment we assume a low dose effect if the MOA is active at low dose and/or is shared by a human disease and/or by a ubiquitous chemical [21]. Based on analysis of MOA studies, we have identified 4 potentially effective low dose effects of chlordecone: cancer promotion, developmental impairment, hepatotoxicity, and neurotoxicity. Only hepatotoxicity is not driven by an endocrine disruption mechanism. ERFs with a linear shape at low dose have been derived for these effects from epidemiological studies (prostate cancer and cognitive development) and animal studies (liver cancer). There are no quantitative data for neurotoxic effects in adulthood. Since the early 80s when damage to DNA appeared to be a major cause of most cancers, non-mutagenic chemicals (such as chlordecone) have been thought unable to generate cancers in human. In a very recent collaborative research, involving almost 200 cancer biologists, examination of MOA found that among 85 non-mutagenic chemicals (not IARC group 1, carcinogens) only 15 % show evidence of a dose–response threshold, whereas 59 % exerted low-dose effects (no dose–response information was found for the remaining 26 %). The authors suggests that the cumulative effects of individual non-carcinogenic chemicals acting on different pathways, and a variety of related systems, organs, tissues and cells could plausibly conspire to produce carcinogenic synergies [139]. Moreover, some endocrine disrupting chemicals are known to have adverse effects at low-dose. Most of them could act on development, fertility, immunity response, or central nervous system. Nonmonotonic dose response seems to be a common hallmark endocrine disrupting chemicals, with stronger response at the low-dose than at higher doses [140]. Thus our findings on chlordecone appear consistent with others involving new approaches based on MOA and weight of evidence analysis.

An additional ERF was derived for kidney damage to allow comparison with the 3 low dose effects. Kidney damage is considered by health and safety agencies as the most sensitive effect. Unfortunately its mechanism of action is not well known. Chlordecone seems to favor the occurrence of SLE (Systemic Lupus Erythematosus) in genetically susceptible animals [10], but this effect is not observed in strains that are not lupus-prone [10]. The underlying mechanism favoring autoimmunity could be an increase of B cells in ways that are not fully comparable with those of a pure estrogen such as estradiol [141]. On the other hand, direct toxicity of chlordecone on renal tissues cannot be excluded [18]. Unfortunately, data from Larson et al. relate to the onset of kidney damage and not to the onset of SLE. Given these shortcomings one can hypothesize that if chlordecone causes kidney damage it is only in individuals prone to SLE.

We have derived 4 ERFs to quantify those effects at low doses of chlordecone, each with a different MOA. Cancers of the liver may be due to the production of reactive oxygen species via induction of CYP450. The other 3 effects are potentially linked to endocrine disruption mechanisms but they affect different organs and different population categories: brain development of boys, prostate cancer in men over 44 years, and renal disease in women. Using these 4 ERFs in the same population therefore cannot produce double counting of damages. An adult can suffer from 2 effects of chlordecone via 2 distinct mechanisms occurring in 2 distinct organs, for men: liver cancer and prostate cancer; for women: liver cancer and kidney damage.

Animal data were modeled as absolute risk (“added risk” in BMDS). In addition all data sets have been modeled with the option of relative risk (“extra risk” in BMDS) for comparison purposes (results not shown). When the incidence in the control group is zero (for example liver cancer in rats of both sexes and in female mice) relative risk and absolute risk BMD_10_ are identical. When the incidence of hepatocellular carcinoma in the control group is not zero, like in male mice, the relative risk BMD_10_ (logistic model: 0.44 mg/kg/d) is less than the absolute risk BMD_10_ (logistic model: 0.54 mg/kg/d). This is an increase of 22 % of the dose giving a 10 % risk. This proportion corresponds to the background incidence in male mice: 21 %. This is also true for kidney damage where the incidence in the control group of female rats is 12 %. The difference between the relative risk BMD_10_ (0.109 mg/kg/d) and the absolute risk BMD_10_ (0.126 mg/kg/d) is 15 %. But since the background incidence of diseases in laboratory animals cannot be considered representative of the background incidence in human population, we recommend using the option of absolute risk when estimating BMD for ERF derivation. Thereby our ERF from animal data is used for humans without the need to know the background incidence.

The ERFs from human studies are based on relative risk and weighted by the background incidence, whereas those from animal studies are based on absolute risk. We wanted to know if the use of relative risk with animal data would change the numerical value of the ERF. This is possible only when the background incidence of the effect is not zero. In the Larson study, the incidence of kidney damage in the female control group was not zero (12 % of kidney damage). So we calculated the relative risks using the R software as in an epidemiological study. ERFs are then derived according to equation 1: ERF = (RR-1)/a. The geometric mean of the 3 ERFs for the 3 dose groups significantly different from the control group was 67 (mg/kg/d)^−1^. If we multiply this ERF by background incidence (I_0_), an absolute risk ERF is obtained (RR-1/a × I_0_ = (I_0_ - I_e_)/a). Here the absolute risk ERF would be 8.04 (mg/kg/d)^−1^ = 67 (mg/kg/d)^−1^ × 12 %. This value is close to the absolute risk ERF obtained through BMD modeling: 7.92 (mg/kg/d)^−1^. If one derives the ERF from the first significant dose group, then the absolute ERF is 10.26 (mg/kg/d)^−1^, with the second group it is 10.74 (mg/kg/d)^−1^ and with the third group 4.72 (mg/kg/d)^−1^. The 2 approaches appear to give comparable results. Conversely, one could derive an ERF from epidemiological data with BMD models, but in the case of chlordecone there is no data for testing this approach. It would require cohort studies because in the case–control studies background incidence is not known. One would also need at least 2 exposure groups in the epidemiological study significantly different from the reference exposure group. The corresponding epidemiological data for these 2 conditions are unusual. In summary the 2 approaches, epidemiological RR or BMD modeling, can be considered as equivalent for ERF derivation.

The study used to derive the ERF for prostate cancer was conducted in Guadeloupe. With over 600 incident cases, it was the largest study on the association between exposure to organochloride pesticides and risk of prostate cancer known at the time of its publication. The multivariate adjustment increases the odds ratios, which is unusual and may indicate the presence of protective cofactors among confounders. The measured increased risk does not include the exposure during the periods of development in utero or of adolescence, critical periods for carcinogenic effects and endocrine disruption. The authors discuss the role of chlordecone as promoter via its affinity for cell estrogenic receptors (ERα), confirmed by our analysis of the MOA. Affinity for this receptor ERα could also promote angiogenesis [117]. The ERF based on this study derives from the highest exposure group because it is the only one that is statistically significant. The ERF from the lower exposure group would be 4 times higher and would estimate 4 times more risk and impacts. However, that would propagate high uncertainty because the lower bound of this RR is less than 1 (meaning less risk in this exposed group than in reference group). Thus, our ERF appears to be of good quality and there are more arguments in favor of an underestimation of risk than in favor of an overestimation.

A strength of the study supporting the ERF for altered cognitive development is its prospective cohort design. However the number of children involved is quite low. Some results are inconsistent, for example for communication skills the slope is negative (deterioration) in the low exposure group and positive (improvement) in the high exposure group; however, those results are not statistically significant. Only the decrease in fine motor skills in boys is statistically significant. The greatest uncertainty related to this effect is not in the results of the study but in the interpretation we have made for the monetary valuation. The measurement of cognitive development levels uses the ASQ scores, but we do not know the monetary value of an ASQ point while we know quite well that of an IQ point. We assume that an ASQ score point at 18 months is equal to an IQ point at age 6–7 years. It is very difficult to know if this assumption overestimates or underestimates the true strength of the effect because there are no relevant studies. To estimate the uncertainty we consider as lower bound that an ASQ point of fine motor skills at 18 months equals 1/5 IQ point at 7 years (only one of 5 ASQ scores is reduced by chlordecone), and as the upper bound we consider that an ASQ point is equal to 2 IQ points (the deterioration of cognitive development suffered in utero continues in the following years since exposure is continuous).

The ERF for liver cancer due to chlordecone derives from an animal study [19]. Another study found increased liver hyperplasia [7] but did not use histological examinations to know if the lesions are carcinogenic or not. The NCI study involves 2 species (mouse and rat), both sexes in each species and 50 animals per dose group. Many other organs were subjected to histological examinations but only the hepatic carcinomas were correlated with chlordecone doses. However, the NCI study has weaknesses already mentioned. The US EPA has used a time to tumor model to estimate BMD_10_ for female rats and mice of both sexes [18]. The geometric mean of 3 BMD_10_ obtained with the time to tumor model is 0.075 mg/kg/d, that of mice only is 0.038 mg/kg/d. By comparison, our results with the time adjustment are: 0.076 mg/kg/d and 0.037 mg/kg/d. In theory the time to tumor model is better but it needs the date of onset of tumors, information that is not always available in publications. Here the 2 approaches give very similar results; this indicates a good robustness of our approach.

The ERF for prostate cancer derives from a recent human study that took into account the cofactors and its variability seems to us properly represented by the 95 % confidence interval of the odds ratio. In the study on impaired cognitive development, the main uncertainty comes from our assumption about the relationship between ASQ scores and IQ points. We used a simple assumption, easily modifiable when better knowledge becomes available. For the ERF derived from animal studies, the main variability comes from either the BMD value or the conversion factor of ingestion dose to blood chlordecone concentration (CF_e/i_). This factor is based on chlordecone pharmacokinetic knowledge that is described in the Additional file 1. The available human data indicate a half-life of blood chlordecone ranging from 63 to 192 days with an average of 127.5 j. The CF_e/i_ obtained from these half-lives are: mean CF_e/i_ = 0.064 (mg/kg/d)/(μg/L); low CF_e/i_ = 0.043 (mg/kg/d)/(μg/L); high CF_e/i_ = 0.131 (mg/kg/d)/(μg/L) (see Additional file 1), numbers we use for framing the variability of the ERF if the range (range = 3.1 = 0.131/0.043) is larger than the range between the smallest and the largest BMD value.

There are only 2 exposure studies before 2003 (INSERM and HIBISCUS) and their enrollment is lower than in the studies after 2003 (KARUPROSTATE and TIMOUN). The representativeness of the INSERM study data for men ages 20 to 45 is uncertain because the participants are workers followed by the occupational medical service. This excluded those who are unable to work, unemployed or independent workers. However these are the only data available before 2004. In addition, one of the objectives of this cross-sectional study was to describe the exposure to chlordecone in adult men. Moreover the age group in the INSERM study (20–45 years) does not match the age group in the KARUPROSTATE study (>44 years). In KARUPROSTATE the control group is a random sample of men aged over 44 years summoned by the national health insurance for free and systematic health screening; a recruitment bias is unlikely. The representativeness of the HIBISCUS study of women of childbearing age also involves uncertainty, but more limited than for INSERM. Recruitment for HIBISCUS was similar to the TIMOUN study: women in the sixth month of pregnancy planning to give birth in a public hospital in Guadeloupe (about 70 % of births). In summary, the exposure data available before 2004 aimed to assess the exposure of men and women of reproductive age. The representativeness of HIBISCUS and especially of INSERM for the general population involves uncertainties that are difficult to estimate. Anyway, these are the only exposure data available in 2013, and they will be used to assess the risks and impacts of exposure to chlordecone in Guadeloupe.

## Conclusions

This is the first study, to our knowledge, to yield explicit exposure-response functions (ERFs) for non-genotoxic effects of chronic low exposure to chlordecone. It is also one of the first studies explicitly designed to implement the recommendations of the North American National Academy of Sciences, described in the Silver Book in 2009 [21].

The methodological framework developed here allows estimating ERFs that produce a central risk estimate. These ERFs are robust with regard to choice of models, thus our methodological framework could be used generally. Furthermore, the ERFs obtained could be used in any population, without need to know the background incidence of a disease. Some ERFs are expressed in blood chlordecone concentration (μg/l), others are expressed in dose by ingestion (chlordecone from food intake). For use in a risk assessment study, it may be necessary to have a conversion factor between ingestion dose and blood levels of chlordecone. In the evaluation of health and economic impacts, the ERF uncertainties can be framed by the lower and upper bounds of each ERF. In any case, these ERF pave the way for a quantitative assessment of risks and impacts for non-mutagens chemicals, a great added value for public health decisions.

## Abbreviations

AIC, Akaïke’s Information Criterion; ASQ, age and stage questionnaire; BMD, benchmark dose; BMD_10_, benchmark dose for an extra risk of 10 %; BMD_HED_, benchmark dose expressed as human equivalent dose; BMR, benchmark response; CF, conversion factor; CYP450, cytochrome P450; DAF, dosimetric adjustment factor; ERF, exposure-response function; ERα, estrogen receptor alpha; IQ, intellectual quotient; LD, Limit of Detection; LOAEL, lowest observed adverse effect level; MLE, maximum likelihood estimation; MOA, mode of action; NCI, National Cancer Institute; NOAEL, no observed adverse effect level; OR, odds ratio; P25, P50, Px, percentile 25, percentile 50, percentile x.; POD, point of departure; RfD, reference dose; RR, relative risk; SLE, systemic lupus erythematosus; US EPA, United State Environmental Protection Agency; WHO, World health Organization; βM, mean of the censured data estimated with β-substitution method

## Additional files

Additional file 1: Figure I. Decrease of chlordecone concentrations in rat organs and internal fluids over a 182 days period of time. **Figure II.** Decrease of blood chlordecone, in humans and in rats, after exposure cessation. **Figure III.** Modeled increase of blood chlordecone in humans and in rats following a continuous daily exposure to 0.5 μg/kg/d. (RTF 11334 kb) Additional file 2: Table A. DAF Calculated with the data of the studies. **Table B.** Data set on liver cancer from NCI 1976 study, and statistical tests. **Table C.** Data set on liver cancer from Reuber 1979 study, and statistical tests. **Table D.** Food consumption per dose group and sex in the Larson 1979 study, and conversion of dose to metric units. **Table E.** Data set on liver hyperplasia from Larson 1979 study, and statistical tests. **Table F.** Data set on hepatotoxicity (hyperplasia and change of liver fat) from Larson 1979 study, and statistical tests. **Table G.** Data set on glomerulosclerosis from Larson 1979 study, and statistical tests. **Table G.** Distribution of blood chlordecone concentration in Guadeloupe by period of time, data extracted from Guldner 2011. **Table I.** Parameters of the β-substitution model for substituted mean value to results < LD in epidemiological studies on chlordecone effects in Guadeloupe. (DOCX 116 kb)
