# Supplementary material for: Public health and chronic low chlordecone exposure in Guadeloupe, Part 1: hazards, exposure-response functions, and exposures
Source: Environ Health. 2016 Jul 12;15:75. doi: 10.1186/s12940-016-0160-x (PMC4942950; doi:10.1186/s12940-016-0160-x)
Supplement: Additional file 1: Figure I. — Decrease of chlordecone concentrations in rat organs and internal fluids over a 182 days period of time. Figure II. Decrease of blood chlordecone, in humans and in rats, after exposure cessation. Figure III. Modeled increase of blood chlordecone in humans and in rats following a continuous daily exposure to 0.5 μg/kg/d. (RTF 11334 kb) [file 12940_2016_160_MOESM1_ESM.rtf]

Public health and chronic low chlordecone exposure in Guadeloupe. Part 1: Hazards, exposure-response functions, and exposure.

Vincent Nedellec1, Ari Rabl2, William Dab3.


Additional file 1


Pharmacokinetic knowledge

As an organochloride pesticide, chlordecone has unusual properties in humans as in animals. The first of these is its high affinity for blood proteins and low affinity for body fat. Then there is a dominant accumulation in the liver at the expense of storage in fat. The third is an inhomogeneous distribution in different tissues or regions of the same organ (especially in the CNS) probably due to variations in pH and protein levels. In humans as in animals, chlordecone enters the body via an effective passive diffusion mechanism. Little of this chemical is metabolized. It is distributed preferentially in descending order to: liver, subcutaneous fat, muscle, bile and blood (no data for other human organs or tissues). In human blood its mean half-life is about 6 months compared to 4 months in fat. Blood clearence is slower in humans than in rats (half-life in rat blood = 2-3 weeks). The decreases are linear in organs and tissues. There is no known threshold in either of these kinetic phases.

Internal distribution of chlordecone in humans
Chlordecone is widely distributed in the body. In workers poisoned at Hopewell (USA), the ratios of concentrations between various tissues or fluids and blood were: liver 15, subcutaneous fat 7, muscles 3, bile 2.5 [1]. Chlordecone preferentially binds to blood proteins: albumin and high molecular weight lipoproteins in humans [2, 3] as well as in animals [4, 5]. This disposition is responsible for its persistence in the blood [6]. It would also explain its privileged accumulation in the liver [1]. Brain tissue levels of chlordecone in humans are not known but presumably they are at least equal to that of the rat. The distribution of chlordecone in the brains of rats is not uniform. It is preferentially concentrated in the striatum and medulla/pons [7]. Another study found concentrations of chlordecone in the nervous system well above the blood concentration and also inhomogeneous area distribution: sciatic nerve > spinal cord > cerebellum > brain [8] (cf. figure A). In humans, the affinity of chlordecone for body fat is half of that in animals. It is in any case unusually low for a hydrophobic substance [6].

Metabolism
The metabolism of chlordecone is low in humans as in animals. It has a ketone which is the cause of its glucuronidation. The chlordecone reductase family of aldo-keto reductase may, prior to glucuronidation, reduce the hydrate chlordecone into chlordecone alcohol. This liver enzyme exists only in humans, gerbils and rabbits [9-15]. In bile from intoxicated workers at Hopewell (USA), 72% of the chlordecone is found in free form, with only a small portion conjugated with glucuronic acid (9%). The remaining fraction of the total chlordecone measured in bile appeared to be a stable polar metabolite resistant to beta-glucuronidase [16]. Thus, if the metabolism of chlordecone could be different from humans to rodents, this difference concerns only a small fraction of the total chlordecone. 

Excretion / Elimination
In humans, the elimination of chlordecone is mainly fecal. It preferably takes the bile pathway with a bile/feces ratio from 10 to 20 [17, 1]. Most of the bile chlordecone is reabsorbed in the gut. The direct intestinal excretion is possible because when the biliary secretion is deflected of intestines, chlordecone is still present in the stool. Only the alcohol glucuronide fraction seems to have disappeared [17]. This is confirmed in the gerbil, mouse and monkey [6]. Fecal excretion of chlordecone in humans is accelerated by the administration of cholestyramine [17, 18, 1 , 19]. A reduced form chlordecone in human faeces is deca-chloro-octahydro-1,3,4-metheno-2H-cyclobuta(c,d)pentalen-2-ol [20].
Breast milk is a major route of excretion for chlordecone. It was found in maternal milk in the USA [21] as well as in Guadeloupe [22].
There are negligible quantities of chlordecone in sweat, saliva and human sebum [1]. 

Differences between humans and animals
There are limited pharmacokinetic differences between humans and other mammals. Only two aspects can be of importance for risk assessment: a greater affinity for the human blood than for animal blood and a lack of chlordecone reductase in rats.
The first aspect strengthens the observed difference in blood chlordecone elimination rate. Thus, if a conversion factor for external dose to internal concentration is to be estimated, it must be based on human blood elimination rate. 
The absence of chlordecone reductase in rats is of lesser importance. First, because only a marginal fraction of the internal chlordecone is concerned and second because other conjugation pathways exist in rats. In addition, another aldo-keto reductase is present in rats: 3 alpha-hydroxysteroid dehydrogenase (3HSD) has the same functional and structural properties as human chlordecone reductase [9, 10, 23]. A DNA sequence coding for the rat 3HSD is identical to that of human encoding chlordecone reductase, and three other sequences share a high degree of similarity [24]. The 3HSD in rat brain produces tétrahydrostéroïdes that interfere with key receptor inhibition of gamma aminobutyric acid. In humans, the 3 genes coding for chlordecone reductase and two dihydrodiols dehydrogenases are located on chromosome 10 at the 10p14-p15 position [15]. In rabbits, a protein with similar functions to chlordecone reductase has been identified in the uterus: the 20 alpha-hydroxysteroid dehydrogenase [25, 23]. In pigs, chlordecone reductase does not exist. However, one study showed that the reduction and conjugation of chlordecone was very similar to that of man [26]. 

Quantitative relationship between external exposure and blood concentration in humans.
Not all the necessary data for the construction of a comprehensive PBPK model in humans are available at the time of this study (2013). The available PBPK models only use animal data [27-29]. In addition, these PBPK models do not always provide the blood concentration for a given external dose. Because of differences in the persistence of chlordecone in blood in humans and animals (Figure B), a new model is built for chronic exposures. It is a simple model with two phases: 1. absorption during the first 24 hours, 2. blood clearance rates. We make the following assumptions: the first phase is linearly correlated with the initial dose, the second is specific and linear in humans (cf. figure B).
There are no known human data for the first phase. There are four animal studies that meet the following criteria: mammalian species, 14C labeled chlordecone, results in numerical values, blood test within 24 hours of the first dosing (gavage or intraperitoneal injection) [8, 30, 29, 31]. The average "external dose / blood level" ratio in these studies was 8.13 (µg/kg)/(µg/l).
With 12 employees of Allied Chemical Corporation (USA, Baltimore), the blood half-life of chlordecone after cessation of exposure was 63-148 days. This decrease does not seem dependent on the level of the initial blood concentration, which would indicate a single-phase kinetics [32]. With 32 employees of Product Life Sciences Corporation (USA, Hopewell), exposed to levels 10-20 times higher than in Baltimore, the blood half-life of chlordecone after cessation of exposure was 165 ± 27 days [1]. Thus, the half-life measured in humans extends from 63 to 192 days. We chose a blood half-life average of 127.5 days. The daily decay rate is 0.39% / day (range [0.26 to 0.79% / day]). The decrease based on the half-life should be mathematically infinite. However, there comes a time when the residual concentration of a single dose approaches zero and becomes marginal. Considering that a linear decrease is a good approximation, then the initial blood concentration should reach the value 0 after a time equal to twice the half-life, here 255 day [126-384 day]. With a daily dose of 0.5 µg/kg/day, one can empirically determine the maximum blood concentration to be 7.76 µg/l [3.81 to 11.71 µg/l] (Figure C). From which we have calculated a conversion factor for external to internal chronic exposure of 0.064 [0.043-0.131] (µg/kg/day)/(µg/l).


Figure I: Decrease of chlordecone concentrations in rat organs and internal fluids over a 182 days period of time.


Figure II: Decrease of blood chlordecone, in humans and in rats, after exposure cessation.


Figure III: Modeled increase of blood chlordecone in humans and in rats following a continuous daily exposure to 0,5 µg/kg/d.


References


1. Cohn WJ, Boylan JJ, Blanke RV, Fariss MW, Howell JR, Guzelian PS. Treatment of chlordecone (Kepone) toxicity with cholestyramine. Results of a controlled clinical trial. The New England journal of medicine. 1978;298(5):243-8. doi:10.1056/NEJM197802022980504.
2. Maliwal BP, Guthrie FE. In vitro uptake and transfer of chlorinated hydrocarbons among human lipoproteins. Journal of lipid research. 1982;23(3):474-9. 
3. Soine PJ, Blanke RV, Guzelian PS, Schwartz CC. Preferential binding of chlordecone to the protein and high density lipoprotein fractions of plasma from humans and other species. Journal of toxicology and environmental health. 1982;9(1):107-18. doi:10.1080/15287398209530146.
4. Skalsky HL, Fariss MW, Blanke RV, Guzelian PS. The role of plasma proteins in the transport and distribution of chlordecone (Kepone) and other polyhalogenated hydrocarbons. Annals of the New York Academy of Sciences. 1979;320:231-7. 
5. Soine PJ, Blanke RV, Chinchilli VM, Schwartz CC. High-density lipoproteins decrease the biliary concentration of chlordecone in isolated perfused pig liver. Journal of toxicology and environmental health. 1984a;14(2-3):319-35. doi:10.1080/15287398409530583.
6. Guzelian PS. The clinical toxicology of chlordecone as an example of toxicological risk assessment for man. Toxicology letters. 1992;64-65 Spec No:589-96. 
7. Fujimori K, Benet H, Mehendale HM, Ho IK. Comparison of brain discrete area distributions of chlordecone and mirex in the mouse. Neurotoxicology. 1982b;3(2):125-29. 
8. Egle JL, Fernandez JB, Guzelian PS, Borzelleca JF. Distribution and excretion of chlordecone (Kepone) in the rat. Drug metabolism and disposition: the biological fate of chemicals. 1978;6(1):91-5. 
9. Binstock JM, Iyer RB, Hamby CV, Fried VA, Schwartz IS, Weinstein BI et al. Human hepatic 3 alpha-hydroxysteroid dehydrogenase: possible identity with human hepatic chlordecone reductase. Biochemical and biophysical research communications. 1992;187(2):760-6. 
10. Deyashiki Y, Tamada Y, Miyabe Y, Nakanishi M, Matsuura K, Hara A. Expression and kinetic properties of a recombinant 3 alpha-hydroxysteroid/dihydrodiol dehydrogenase isoenzyme of human liver. Journal of biochemistry. 1995;118(2):285-90. 
11. Molowa DT, Shayne AG, Guzelian PS. Purification and characterization of chlordecone reductase from human liver. The Journal of biological chemistry. 1986b;261(27):12624-7. 
12. Molowa DT, Wrighton SA, Blanke RV, Guzelian PS. Characterization of a unique aldo-keto reductase responsible for the reduction of chlordecone in the liver of the gerbil and man. Journal of toxicology and environmental health. 1986a;17(4):375-84. doi:10.1080/15287398609530832.
13. Winters CJ, Molowa DT, Guzelian PS. Isolation and characterization of cloned cDNAs encoding human liver chlordecone reductase. Biochemistry. 1990;29(4):1080-7. 
14. Houston TE, Mutter LC, Blanke RV, Guzelian PS. Chlordecone alcohol formation in the Mongolian gerbil (Meriones unguiculatus): a model for human metabolism of chlordecone (kepone). Fundamental and applied toxicology : official journal of the Society of Toxicology. 1981;1(3):293-8. 
15. Khanna M, Qin KN, Klisak I, Belkin S, Sparkes RS, Cheng KC. Localization of multiple human dihydrodiol dehydrogenase (DDH1 and DDH2) and chlordecone reductase (CHDR) genes in chromosome 10 by the polymerase chain reaction and fluorescence in situ hybridization. Genomics. 1995b;25(2):588-90. 
16. Fariss MW, Blanke RV, Saady JJ, Guzelian PS. Demonstration of major metabolic pathways for chlordecone (kepone) in humans. Drug metabolism and disposition: the biological fate of chemicals. 1980;8(6):434-8. 
17. Boylan JJ, Cohn WJ, Egle JL, Jr., Blanke RV, Guzelian PS. Excretion of chlordecone by the gastrointestinal tract: evidence for a nonbiliary mechanism. Clinical pharmacology and therapeutics. 1979;25(5 Pt 1):579-85. 
18. Boylan JJ, Egle JL, Guzelian PS. Cholestyramine: Use as a new therapeutic approach for chlordecone (Kepone) poisoning. Science. 1978b;199(4331):893-5. 
19. Guzelian PS. Therapeutic approaches for chlordecone poisoning in humans. J Toxicol Environ Health. 1981;8:757-66. 
20. Blanke RV, Fariss MW, Guzelian PS, Patterson AR, Smith DE. Identification of a reduced form of chlordecone (Kepone) in human stool. Bull Environ Contam Toxicol. 1978;20(6):782-5. 
21. Suta BE. Human population exposures to mirex and Kepone. US NTIS PS Rep PS. 1979;790(109). 
22. Boucher O, Simard MN, Muckle G, Rouget F, Kadhel P, Bataille H et al. Exposure to an organochlorine pesticide (chlordecone) and development of 18-month-old infants. Neurotoxicology. 2013;35C:162-8. doi:10.1016/j.neuro.2013.01.007.
23. Lin HK, Hung CF, Moore M, Penning TM. Genomic structure of rat 3alpha-hydroxysteroid/dihydrodiol dehydrogenase (3alpha-HSD/DD, AKR1C9). The Journal of steroid biochemistry and molecular biology. 1999;71(1-2):29-39. 
24. Khanna M, Qin KN, Cheng KC. Distribution of 3 alpha-hydroxysteroid dehydrogenase in rat brain and molecular cloning of multiple cDNAs encoding structurally related proteins in humans. The Journal of steroid biochemistry and molecular biology. 1995a;53(1-6):41-6. 
25. Lacy WR, Washenick KJ, Cook RG, Dunbar BS. Molecular cloning and expression of an abundant rabbit ovarian protein with 20 alpha-hydroxysteroid dehydrogenase activity. Molecular endocrinology. 1993;7(1):58-66. 
26. Soine PJ, Blanke RV, Schwartz CC. Chlordecone metabolism in the pig. Toxicology letters. 1983;17(1-2):35-41. 
27. Belfiore CJ, Yang RS, Chubb LS, Lohitnavy M, Lohitnavy OS, Andersen ME. Hepatic sequestration of chlordecone and hexafluoroacetone evaluated by pharmacokinetic modeling. Toxicology. 2007;234(1-2):59-72. 
28. Bungay PM, Dedrick RL, Matthews HB. Pharmacokinetics of environmental contaminants. In: Dynamics, Exposure and Hazard Assessment of Toxic Chemicals Haque, R, ed. 1980. 
29. Bungay PM, Dedrick RL, Matthews HB. Enteric transport of chlordecone (Kepone) in the rat. J Pharmacokinet Biopharm. 1981;9(3):309-42. 
30. Kavlock R, Chernoff N, Rogers E, Whitehouse D. Comparative tissue distribution of mirex and chlordecone in fetal and neonatalrats. Pesticide Biochemistry and Physiology. 1980;14(3):227-35. 
31. Lee J, Scheri RC, Curtis LR. Chlordecone altered hepatic disposition of [14C]cholesterol and plasma cholesterol distribution but not SR-BI or ABCG8 proteins in livers of C57BL/6 mice. Toxicol Appl Pharmacol. 2008b;229(3):265-72. 
32. Adir J, Caplan YH, Thompson BC. Kepone serum half-life in humans. Life sciences. 1978;22(8):699-702. 
