# Supplementary material for: Public health and chronic low chlordecone exposure in Guadeloupe, Part 1: hazards, exposure-response functions, and exposures
Source: Environ Health. 2016 Jul 12;15:75. doi: 10.1186/s12940-016-0160-x (PMC4942950; doi:10.1186/s12940-016-0160-x)
Supplement: Additional file 2: Table A. — DAF Calculated with the data of the studies. Table B. Data set on liver cancer from NCI 1976 study, and statistical tests. Table C. Data set on liver cancer from Reuber 1979 study, and statistical tests. Table D. Food consumption per dose group and sex in the Larson 1979 study, and conversion of dose to metric units. Table E. Data set on liver hyperplasia from Larson 1979 study, and statistical tests. Table F. Data set on hepatotoxicity (hyperplasia and change of liver fat) from Larson 1979 study, and statistical tests. Table G. Data set on glomerulosclerosis from Larson 1979 study, and statistical tests. Table G. Distribution of blood chlordecone concentration in Guadeloupe by period of time, data extracted from Guldner 2011. Table I. Parameters of the β-substitution model for substituted mean value to results < LD in epidemiological studies on chlordecone effects in Guadeloupe. (DOCX 116 kb) [file 12940_2016_160_MOESM2_ESM.docx]

**Public health and chronic low chlordecone exposure in Guadeloupe. Part 1: Hazards, exposure-response functions, and exposure.**

**Vincent Nedellec^1^, Ari Rabl^2^, William Dab^3^.**

**Additional file 2**

**List of tables**

[Table A: DAF calculated with the data of the studies. 1](#_Toc448310067)

[Table B: Data sets for liver cancer from NCI (1976) [2], and statistical tests 2](#_Toc448310068)

[Table C: Data sets for liver cancer from Reuber (1979) [3], and statistical tests 3](#_Toc448310069)

[Table D: Food consumption per dose groups and sex in Larson (1979) [1], and conversion of dose to metric units 4](#_Toc448310070)

[Table E: Data sets for liver hyperplasia from Larson (1979) [1], and statistical tests 5](#_Toc448310071)

[Table F: Data sets for hepatotoxicity (hyperplasia + fatty change) from Larson (1979) [1], and statistical tests 6](#_Toc448310072)

[Table G: Data sets for glomeruloschlerosis from Larson (1979) [1], and statistical tests 7](#_Toc448310073)

[Table H: Blood chlordecone concentrations distribution in Guadeloupe by period of time (data from Guldner 2011 [4]). 8](#_Toc448310074)

[Table I: Parameters of the β-substitution model for estimate a βM in exposure measurements that are <LD 9](#_Toc448310075)

Table A: DAF calculated with the data of the studies.

| **Studies** | **Species** | **sex** | **n** | **Animal BW (kg)** | **Human BW (kg)** | **DAF** | **Unisex DAF weighted average** |
| --- | --- | --- | --- | --- | --- | --- | --- |
| Larson 1979 [1] | Rat Wistar | F | 80 | 0.297 | 70 | **0.255** | **0.267** |
| Larson 1979 [1] | Rat Wistar | M | 52 | 0.462 | 70 | **0.285** |  |
| NCI 1976 [2] | Rat Osborne-Mendel | F | 204 | 0.389 | 70 | **0.273** | **0.283** |
| NCI 1976 [2] | Rat Osborne-Mendel | M | 209 | 0.514 | 70 | **0.293** |  |
| NCI 1976 [2] | Mice B6C3F1 | F | 149 | 0.0353 | 70 | **0.150** | **0.151** |
| NCI 1976[2] | Mice B6C3F1 | M | 165 | 0.0373 | 70 | **0.152** |  |

DAF = dosimetric adjustment factor = (animal BW/human BW)^1/4^

Unisex DAF weighted average = {(DAF_F_ * n_F_ ) + (DAF_M_ * n_M_)} / ( n_F_+n_M_), used if BMD is estimated with grouped data of female and male.

Table B: Data sets for liver cancer from NCI (1976) [2], and statistical tests

|  | **Animal doses* mg/kg/d (TWA)** | **HED doses** mg/kg/d** | **n** | **healthy** | **Liver carcinomas** | **Incidence (%)** | **Expected healthy (E_h_)** | **Expected (E_c_) case** | **Cochran-Armitage Trend Test** | **Fisher's exact test if one E_i_ <3 p-value** | **χ²  if  all E_i_ ≥ 5** | **Yates' χ² if  2< E_i_ <5** | **p-value for χ²** |
| --- | --- | --- | --- | --- | --- | --- | --- | --- | --- | --- | --- | --- | --- |
| **Female Rat Osborne-Mendel** | | | | | | | | | | | | | |
| Control (pooled + matched) | 0 | 0.0 | 110 | 110 | 0 | 0% | 104.1 | 5.9 | **Z=4.688  p<0.0001** |  |  |  |  |
| Low dose (TWA=18ppm) | 0.94 | 0.3 | 49 | 48 | 1 | 2% | 46.4 | **2.6** |  | **0.308** |  |  |  |
| High dose (TWA=26ppm) | 1.4 | 0.4 | 45 | 35 | 10 | 22% | 42.6 | **2.4** |  | **1.95E-06** |  |  |  |
| All |  |  | 204 | 193 | 11 |  |  |  |  |  |  |  |  |
| **Male Rat Osborne-Mendel** | | | | | | | | | | | | | |
| Control (pooled + matched) | 0 | 0.0 | 115 | 115 | **0** | 0% | 112.8 | **2.2** | **Z=2.806  p<0.0025** |  |  |  |  |
| Low dose(TWA=8ppm) | 0.36 | 0.11 | 50 | 49 | 1 | 2% | 49.0 | **1.0** |  | **0.303** |  |  |  |
| High dose(TWA=24ppm) | 1.10 | 0.32 | 44 | 41 | 3 | 7% | 43.2 | **0.8** |  | **0.020** |  |  |  |
| All |  |  | 209 | 205 | 4 |  |  |  |  |  |  |  |  |
| **Female Mice B6C3F1** | | | | | | | | | | | | | |
| Control | 0 | 0 | 50 | 50 | 0 | 0% | 33.6 | 16.4 | **Z=4.99  p<0.0001** |  |  |  |  |
| Low dose (TWA=20ppm) | 2.9 | 0.43 | 50 | 24 | 26 | 52% | 33.6 | 16.4 |  |  | **35.14** |  | **3.1E-09** |
| High dose (TWA=40ppm) | 5.8 | 0.87 | 49 | 26 | 23 | 47% | 32.9 | 16.1 |  |  | **30.57** |  | **3.2E-08** |
| All |  |  | 149 | 100 | 49 |  |  |  |  |  |  |  |  |
| **Male Mice B6C3F1** | | | | | | | | | | | | | |
| Control | 0 | 0 | 68 | 54 | 14 | 21% | 28.4 | 39.6 | **Z=8.215  p<0.0001** |  |  |  |  |
| Low dose (TWA=20ppm) | 2.9 | 0.44 | 48 | 9 | 39 | 81% | 20.1 | 27.9 |  |  | **41.73** |  | **1.0E-10** |
| High dose (TWA=23ppm) | 3.4 | 0.52 | 49 | 6 | 43 | 88% | 20.5 | 28.5 |  |  | **51.43** |  | **7.4E-13** |
| All |  |  | 165 | 69 | 96 |  |  |  |  |  |  |  |  |

Data in highlighted frame (grey, red and green colored) are calculated; all others data are taken directly from publication. Green highlighted results are statistically significant, red highlighted are not.

Cochran-Armitage Trend Test is calculated with BMDS2.4.0. Fisher's exact and chi-square tests are calculated with “R”. Example formula « fisher.test(matrix(c(110,48,0,1),2,2, byrow=TRUE)) » p-value = 0.308;

chisq.test(matrix(c(50,24,0,26),2,2, byrow=TRUE, correct=FALSE) χ² = 35.14; for Yates’ correction, same formula as chi-square but “correct=TRUE”

*Animal dose have been estimated by USEPA with TOX_RISK computer program {USEPA, 2009 #18}

**HED doses are animal doses multiplied by DAF (see Table A);

Table C: Data sets for liver cancer from Reuber (1979) [3], and statistical tests

|  | **animal dose mg/kg/d** | **n** | **healthy** | **Liver tumor** | **Incidence (%)** | **Expected healthy (E_h_)** | **Expected (E_c_) case** | **Cochran-Armitage Trend Test** | **Fisher's exact test if one E_i_ <3 p-value** | ***R test formula*** |
| --- | --- | --- | --- | --- | --- | --- | --- | --- | --- | --- |
| **Albinos Rats Male (unspecified strain)** | | | | | | | | | | |
| Control | 0 | 8 | 8 | **0** | 0% | 6.5 | **1.5** | **Z=1.601 P=0.0547** |  |  |
| group 1 | 0.25 | 8 | 6 | 2 | 25% | 6.5 | **1.5** |  | **0.4667** | *fisher.test(matrix(c(8,6,0,2),2,2, byrow=TRUE))* |
| group 2 | 0.5 | 10 | 8 | 2 | 20% | 8.1 | **1.9** |  | **0.477** | *fisher.test(matrix(c(8,8,0,2),2,2, byrow=TRUE))* |
| group 3 | 1.25 | 5 | 3 | 2 | 40% | 4.0 | **1.0** |  | **0.128** | *fisher.test(matrix(c(8,3,0,2),2,2, byrow=TRUE))* |
| All |  | 31 | 25 | 6 |  |  |  |  |  |  |
| **Albinos Rats Female (unspecified strain)** | | | | | | | | | | |
| Control | 0 | 18 | 18 | **0** | 0% | 14.5 | **3.5** | **Z=2.802 P=0.0025** |  |  |
| group 1 | 0.25 | 16 | 15 | 1 | 6% | 12.9 | **3.1** |  | **0.4706** | *fisher.test(matrix(c(18,15,0,1),2,2, byrow=TRUE))* |
| group 2 | 0.5 | 15 | 13 | 2 | 13% | 12.1 | **2.9** |  | **0.199** | *fisher.test(matrix(c(18,13,0,2),2,2, byrow=TRUE))* |
| group 3 | 1.25 | 5 | 3 | 2 | 40% | 4.0 | **1.0** |  | **0.040** | *fisher.test(matrix(c(18,3,0,2),2,2, byrow=TRUE))* |
| All |  | 54 | 49 | 5 |  |  |  |  |  |  |

Data in highlighted frame (grey, red and green colored) are calculated; all others data are taken directly from publication. Green highlighted results are statistically significant, red highlighted are not.

Cochran-Armitage Trend Test is calculated with BMDS2.4.0. Fisher's exact and chi-square tests are calculated with “R”.

Table D: Food consumption per dose groups and sex in Larson (1979) [1], and conversion of dose to metric units

| **Time of measurements (weeks)** | **Duration (days)** | **Males** | | | | | **Female** | | | | |
| --- | --- | --- | --- | --- | --- | --- | --- | --- | --- | --- | --- |
|  |  | **Group**  **0 ppm** | **Group**  **1 ppm** | **Group**  **5 ppm** | **Group**  **10 ppm** | **Group**  **25 ppm** | **Group**  **0 ppm** | **Group**  **1 ppm** | **Group**  **5 ppm** | **Group**  **10 ppm** | **Group**  **25 ppm** |
| 5 | 35 | 75 | 75 | 77 | 80 | 85 | 80 | 80 | 83 | 79 | 87 |
| 13 | 56 | 50 | 50 | 54 | 56 | 57 | 56 | 56 | 58 | 54 | 64 |
| 26 | 91 | 36 | 36 | 38 | 43 | 50 | 45 | 45 | 50 | 54 | 62 |
| 52 | 182 | 34 | 34 | 36 | 38 | 49 | 45 | 45 | 50 | 52 | 65 |
| 104 | 364 | 36 | 36 | 54 | 50 | 57 | 39 | 39 | 44 | 56 | 81 |
| **TWA food consumption* (g_food_/kg_BW_/d)** | | 38.5 | 38.5 | 48.6 | 48.0 | 55.5 | 44.5 | 44.5 | 49.2 | 55.7 | 73.6 |
| **Doses (mg/kg/d)** | | **0.00** | **0.04** | **0.24** | **0.48** | **1.39** | **0.00** | **0.04** | **0.25** | **0.56** | **1.84** |

Food consumption are expressed in grams of food per kg of body weight per day (g_food_/kg_BW_/d)

* Time Weighted Average food consumption = (∑ (food consumption × days)) / 728 d

** Doses are calculated as: TWA food consumption × X ppm × 10^-3^ g/mg

Table E: Data sets for liver hyperplasia from Larson (1979) [1], and statistical tests

|  | **animal doses* mg/kg/d** | **HED doses** mg/kg/d** | **n** | **healthy** | **Hyperplasia** | **Incidence (%)** | **Expected healthy (E_h_)** | **Expected (E_c_) case** | **Cochran-Armitage Trend Test** | **Fisher's exact test if one E_i_ <3 p-value** | **R formula** |
| --- | --- | --- | --- | --- | --- | --- | --- | --- | --- | --- | --- |
| **Female Rats (Wistar)** | | | | | | | | | | |  |
| Control | 0.00 | 0.00 | 34 | 34 | 0 | 0% | 32.7 | **1.3** | **Z = 3.167 p<0.0008** |  |  |
| group 1ppm | 0.04 | 0.01 | 13 | 13 | 0 | 0% | 12.5 | **0.5** |  | **1** | *fisher.test(matrix(c(34,13,0,0),2,2, byrow=TRUE))* |
| group 5ppm | 0.25 | 0.06 | 17 | 17 | 0 | 0% | 16.3 | **0.7** |  | **1** | *fisher.test(matrix(c(34,17,0,0),2,2, byrow=TRUE))* |
| group 10ppm | 0.56 | 0.14 | 12 | 12 | 3 | 25% | 11.5 | **0.5** |  | **0.01449** | *fisher.test(matrix(c(34,9,0,3),2,2, byrow=TRUE))* |
| group 25ppm | 1.84 | 0.47 | 4 | 3 | 1 | 25% | 3.8 | **0.2** |  | **0.105** | *fisher.test(matrix(c(34,3,0,1),2,2, byrow=TRUE))* |
| All |  |  | 80 | 78 | 2 |  |  |  |  |  |  |
| **Male Rats (Wistar)** | | | | | | | | | | |  |
| referent | 0.00 | 0.00 | 22 | 22 | 0 | 0% | 21.2 | **0.8** | **Z = 2.556 p<0.0053** |  |  |
| group 1ppm | 0.04 | 0.01 | 11 | 11 | 0 | 0% | 10.6 | **0.4** |  | **1** | *fisher.test(matrix(c(22,11,0,0),2,2, byrow=TRUE))* |
| group 5ppm | 0.24 | 0.07 | 6 | 6 | 0 | 0% | 5.8 | **0.2** |  | **1** | *fisher.test(matrix(c(22,6,0,0),2,2, byrow=TRUE))* |
| group 10ppm | 0.48 | 0.14 | 9 | 7 | 0 | 0% | 8.7 | **0.3** |  | **1** | *fisher.test(matrix(c(22,7,0,0),2,2, byrow=TRUE))* |
| group 25ppm | 1.39 | 0.40 | 4 | 2 | 2 | 50% | 3.8 | **0.2** |  | **0.018** | *fisher.test(matrix(c(22,3,0,2),2,2, byrow=TRUE))* |
| All |  |  | 52 | 49 | 3 |  |  |  |  |  |  |

Data in highlighted frame (grey, red and green colored) are calculated; all others data are taken directly from publication. Green highlighted results are statistically significant, red highlighted are not.

Cochran-Armitage Trend Test is calculated with BMDS2.4.0. Fisher's exact and chi-square tests are calculated with “R”.

*Animal dose have been estimated by us (see Table 5)

**HED doses are animal doses multiplied by DAF (see Table 1)

Table F: Data sets for hepatotoxicity (hyperplasia + fatty change) from Larson (1979) [1], and statistical tests

|  | **animal doses* mg/kg/d** | **HED dose** mg/kg/d** | **n** | **healthy** | **Hepatotoxicity** | **Incidence (%)** | **Expected healthy (E_h_)** | **Expected case (E_c_)** | **Cochran-Armitage Trend Test** | **Fisher's exact test if one E_i_ <3 p-value** | **R formula** |
| --- | --- | --- | --- | --- | --- | --- | --- | --- | --- | --- | --- |
| **Female Rats (Wistar)** | | | | | | | | | | |  |
| Control | 0.00 | 0.00 | 34 | 32 | 2 | 6% | 28.1 | 5.9 | **Z = 1.833 p<0.0334** |  |  |
| group 1ppm | 0.04 | 0.01 | 13 | 12 | 1 | 8% | 10.8 | **2.3** |  | **1** | *fisher.test(matrix(c(32,12,2,1),2,2, byrow=TRUE))* |
| group 5ppm | 0.25 | 0.06 | 17 | 15 | 2 | 12% | 14.1 | **2.9** |  | **0.593** | *fisher.test(matrix(c(32,15,2,2),2,2, byrow=TRUE))* |
| group 10ppm | 0.56 | 0.14 | 12 | 8 | 4 | 33% | 9.9 | **2.1** |  | **0.033** | *fisher.test(matrix(c(32,8,2,4),2,2, byrow=TRUE))* |
| group 25ppm | 1.84 | 0.47 | 4 | 3 | 1 | 25% | 3.3 | **0.7** |  | **0.291** | *fisher.test(matrix(c(32,3,2,1),2,2, byrow=TRUE))* |
| all together |  |  | 80 | 70 | 10 |  |  |  |  | 0.110 | *fisher.test(matrix(c(32,2,12,1,15,2,8,4,3,1),5,2, byrow=TRUE))* |
| **Male Rats (Wistar)** | | | | | | | | | | |  |
| Control | 0.00 | 0.00 | 22 | 21 | 1 | 5% | 18.2 | **3.8** | **Z = 3.488 p<0.0002** |  |  |
| group 1ppm | 0.04 | 0.01 | 11 | 10 | 1 | 9% | 9.1 | **1.9** |  | **1** | *fisher.test(matrix(c(21,10,1,1),2,2, byrow=TRUE))* |
| group 5ppm | 0.24 | 0.07 | 6 | 4 | 2 | 33% | 5.0 | **1.0** |  | **0.107** | *fisher.test(matrix(c(21,4,1,2),2,2, byrow=TRUE))* |
| group 10ppm | 0.48 | 0.14 | 9 | 7 | 2 | 22% | 7.4 | **1.6** |  | **0.195** | *fisher.test(matrix(c(21,7,1,2),2,2, byrow=TRUE))* |
| group 25ppm | 1.39 | 0.40 | 4 | 1 | 3 | 75% | 3.3 | **0.7** |  | **0.006** | *fisher.test(matrix(c(21,1,1,3),2,2, byrow=TRUE))* |
| all together |  |  | 52 | 43 | 9 |  |  |  |  |  |  |

Data in highlighted frame (grey, red and green colored) are calculated; all others data are taken directly from publication. Green highlighted results are statistically significant, red highlighted are not.

Cochran-Armitage Trend Test is calculated with BMDS2.4.0. Fisher's exact and chi-square tests are calculated with “R”.

Table G: Data sets for glomeruloschlerosis from Larson (1979) [1], and statistical tests

|  | **Animal doses* mg/kg/d (TWA)** | **HED doses** mg/kg/d** | **n** | **healthy** | **Glomeruloschlerosis** | **Incidence (%)** | **Expected healthy (E_h_)** | **Expected (E_c_) case** | **Cochran-Armitage Trend Test** | **Fisher's exact test if one E_i_ <3 p-value** | **χ²  if  all E_i_ ≥ 5** | **p-value for χ²** | **R formula** |
| --- | --- | --- | --- | --- | --- | --- | --- | --- | --- | --- | --- | --- | --- |
| **Female rat (Wistar)** | | | | | | | | | | | | |  |
| control group | 0.00 | 0.00 | 34 | 30 | 4 | 12% | 23.4 | 10.6 | Z=3.576 p=0.0002 |  |  |  |  |
| group 1 | 0.04 | 0.01 | 13 | 11 | 2 | 15% | 8.9 | 4.1 |  | 1 |  |  | *fisher.test(matrix(c(30,11,4,2),2,2, byrow=TRUE))* |
| group 2 | 0.25 | 0.06 | 17 | 9 | 8 | 47% | 11.7 | 5.3 |  | 0.01154 | 7.8462 | 0.005 | *fisher.test(matrix(c(30,9,4,8.),2,2, byrow=TRUE))* |
| group 3 | 0.56 | 0.14 | 12 | 4 | 8 | 67% | 8.3 | 3.7 |  | 0.00062 |  |  | *fisher.test(matrix(c(30,4,4,8.),2,2, byrow=TRUE))* |
| group 4 | 1.84 | 0.47 | 4 | 1 | 3 | 75% | 2.8 | 1.2 |  | 0.01517 |  |  | *fisher.test(matrix(c(30,1,4,3),2,2, byrow=TRUE))* |
| **all** |  |  | 80 | 55 | 25 |  |  |  |  |  |  |  |  |
| **Male rat (Wistar)** | | | | | | | | | | | | |  |
| control group | 0.00 | 0.00 | 22 | 10 | 12 | 55% | 10.2 | **11.8** | Z=1.316 p=0.094 |  |  |  |  |
| group 1 | 0.04 | 0.01 | 11 | 8 | 3 | 27% | 5.1 | **5.9** |  | 0.2659 | 2.2 | 0.14 | *fisher.test(matrix(c(10,8,12,3),2,2, byrow=TRUE))* |
| group 2 | 0.24 | 0.07 | 6 | 2 | 4 | 66% | 2.8 | **3.2** |  | 0.6730 |  |  | *fisher.test(matrix(c(10,2,12,4),2,2, byrow=TRUE))* |
| group 3 | 0.48 | 0.14 | 9 | 3 | 6 | 67% | 4.2 | **4.8** |  | 0.6961 | 0.3854 | 0.535 | *fisher.test(matrix(c(10,3,12,6),2,2, byrow=TRUE))* |
| group 4 | 1.39 | 0.40 | 4 | 1 | 3 | 75% | 1.8 | **2.2** |  | 0.6137 |  |  | *fisher.test(matrix(c(10,1,12,3),2,2, byrow=TRUE))* |
| **all** |  |  | 52 | 24 | 28 |  |  |  |  |  |  |  |  |
| **male control group incidence against female control group incidence** | | | | | | | | | | | 11.98 | 5.38E-04 | chisq.test(matrix(c(30,10,4,12),2,2, byrow=TRUE), correct=FALSE) |

Data in highlighted frame (grey, red and green colored) are calculated; all others data are taken directly from publication. Green highlighted results are statistically significant, red highlighted are not.

Cochran-Armitage Trend Test is calculated with BMDS2.4.0. Fisher's exact and chi-square tests are calculated with “R”.

Table H: Blood chlordecone concentrations distribution in Guadeloupe by period of time (data from Guldner 2011 [4]).

| Study Name | Blood samples time period | Population | Age (years) | n | LD | %  < LD | βM for sample <LD (µg/L) | P25 (µg/L) | P50 (µg/L) | P75 (µg/L) | max (µg/L) |
| --- | --- | --- | --- | --- | --- | --- | --- | --- | --- | --- | --- |
| HIBISCUS | 2003 | Mother | 17-45 | 112 | 0.5 | 13 % | 0.321 | 1.2 | 2.2 | 3.9 | 16.6 |
| HIBISCUS | 2003 | Newborn | 0 | 109 | 0.5 | 39 % | 0.394 |  | 0.7 | 1.2 | 3.7 |
| INSERM | 1999-2001 | Men (not farmer) | 20-45 | 45 | 1.5 | 9 % | 1.064 | 2.3 | 5.5 | 9 | 24.3 |
| TIMOUN | 2004-2007 | Mother | 17-45 | 371 | 0.25 | 38 % | 0.182 |  | 0.4 | 0.9 | 19.3 |
| TIMOUN | 2004-2007 | Newborn | 0 | 265 | 0.25 | 72 % | 0.188 |  |  | 0.3 | 22.9 |
| KARUPROSTATE | 2005-2006 | Men (control) | >45 | 406 | 0.25 | 33 % | 0.167 | 0.3 | 0.5 | 1.3 | 44.4 |

βM is calculated by us with the β-substitution method from Ganser and Hewett 2010.

Table I: Parameters of the β-substitution model for estimate a βM in exposure measurements that are <LD

| **Parameters** | **description** | **HIBISCUS***  **(2003)**  **Mothers** | **HIBISCUS***  **(2003)**  **Newborn** | **TIMOUN***  **(2004-2007)**  **Mothers** | **TIMOUN***  **(2004-2007)**  **Newborn** | **INSERM***  **(1999-2001)**  **Men 20-45** | **KARUPROSTATE***  **(2004-2007)**  **Men >44** |
| --- | --- | --- | --- | --- | --- | --- | --- |
| **LD (µg/l)** | Analytical limit of detection | 0.5 | 0.5 | 0.25 | 0.25 | 1.5 | 0.25 |
| **Mean** (µg/l)** | Mean value of blood chlordecone concentrations | 2.2 | 0.7 | 0.4 | 0.3 | 5.5 | 0.5 |
| **n** | Total number of samples | 112 | 109 | 371 | 265 | 45 | 406 |
| **% <LD** | Percentage of samples < LD | 13% | 39% | 38% | 72% | 9% | 33% |
| **k** | Number of samples < LD | 14 | 42 | 140 | 190 | 4 | 133 |
| **βM (µg/l)** | Substitute value for samples < LD | 0.321 | 0.394 | 0.182 | 0.188 | 1.064 | 0.167 |

* The data from: IBISCUS, TIMOUN, INSERM and KARUPROSTAT studies come from Gulner, 2011 [4]

** If the mean is not available, the median is used instead of the mean value.

**References**

1. Larson PS, Egle JL, Jr., Hennigar GR, Lane RW, Borzelleca JF. Acute, subchronic, and chronic toxicity of chlordecone. Toxicol Appl Pharmacol. 1979;48(1):29-41.

2. NCI. Report on carcinogenesis bioassay of technical grade chlordecone(Kepone) CAS No. 143-50-0: U.S. Departement of health, education and welfare, Public Health Service, National Institut of Health 1976.

3. Reuber MD. Carcinomas of the liver in rats ingesting kepone. Neoplasma. 1979;26(2):231-5.

4. Guldner L, Seurin S, Héraud F, Multignier L. Exposition de la population antillaise au chlordécone. BEH. 2011;Numéro thématique – Chlordécone aux Antilles : bilan actualisé des risques sanitaires. (3-4-5):25-8.
